# Design and Analysis of a Single-Camera Omnistereo Sensor for Quadrotor Micro Aerial Vehicles (MAVs) †

**DOI:** 10.3390/s16020217

**Published:** 2016-02-06

**Authors:** Carlos Jaramillo, Roberto G. Valenti, Ling Guo, Jizhong Xiao

**Affiliations:** 1 Deptartment of Computer Science, The Graduate Center, The City University of New York (CUNY), 365 Fifth Avenue, New York, NY 10016, USA; cjaramillo@gradcenter.cuny.edu; 2 Electrical Engineering Department, The City College, City University of New York (CUNY City College), Convent Ave & 140th Street, New York, NY 10031, USA; rvalenti@ccny.cuny.edu; 3 Automation Department, Nanjing University of Science and Technology (NUST), Nanjing 210094, China; laura0955@live.cn

**Keywords:** catadioptrics, omnistereo, 3D perception, Micro Aerial Vehicles (MAVs)

## Abstract

We describe the design and 3D sensing performance of an omnidirectional stereo (omnistereo) vision system applied to Micro Aerial Vehicles (MAVs). The proposed omnistereo sensor employs a monocular camera that is co-axially aligned with a pair of hyperboloidal mirrors (a vertically-folded catadioptric configuration). We show that this arrangement provides a compact solution for omnidirectional 3D perception while mounted on top of propeller-based MAVs (not capable of large payloads). The theoretical single viewpoint (SVP) constraint helps us derive analytical solutions for the sensor’s projective geometry and generate SVP-compliant panoramic images to compute 3D information from stereo correspondences (in a truly synchronous fashion). We perform an extensive analysis on various system characteristics such as its size, catadioptric spatial resolution, field-of-view. In addition, we pose a probabilistic model for the uncertainty estimation of 3D information from triangulation of back-projected rays. We validate the projection error of the design using both synthetic and real-life images against ground-truth data. Qualitatively, we show 3D point clouds (dense and sparse) resulting out of a single image captured from a real-life experiment. We expect the reproducibility of our sensor as its model parameters can be optimized to satisfy other catadioptric-based omnistereo vision under different circumstances.

## 1. Introduction

Micro aerial vehicles (MAVs), such as quadrotor helicopters, are popular platforms for unmanned aerial vehicle (UAV) research due to their structural simplicity, small form factor, vertical take-off and landing (VTOL) capability, and high omnidirectional maneuverability. In general, UAVs have plenty of military and civilian applications, such as target localization and tracking, 3-dimensional (3D) mapping, terrain and infrastructural inspection, disaster monitoring, environmental and traffic surveillance, search and rescue, deployment of instrumentation, and cinematography, among other uses. However, MAVs have size, payload, and on-board computation limitations, which involve the use of compact and lightweight sensors. The most commonly used perception sensors on MAVs are laser scanners and cameras in various configurations such as monocular, stereo, or omnidirectional. We present a vision-based omnidirectional stereo (omnistereo) sensor motivated by several aspects of MAV robotics.

### 1.1. Sensor Motivation

We justify the need for the proposed omnistereo sensor after observing two basic differences in the sensor requirements between MAVs and ground vehicles:

1. Size and payload—In MAV applications, the sensor’s physical dimensions and weight are always a great concern due to payload constraints. Generally, MAVs require fewer and lighter sensors that are compactly designed, while larger robots (including high-payload UAVs) have greater freedom of sensor choice.
2. Field-of-view (FOV)—Due to their omnidirectional motion model, MAVs require a simultaneous observation of the 3D surroundings. Conversely, most ground robots can safely rely upon narrow vision as their motion control on the plane is more stable.

### 1.2. Existing Range Sensors for MAVs

In addition to specifying our sensor requirements, it is important to note the most prevalent robot range sensors used today by MAVs and their limitations. For example, lightweight 2.5D laser scanners can accurately measure distances at fast rates, however, their instantaneous sensing is limited to plane sweeps, which in turn require the quadrotor to move vertically in order to generate 3D maps or to foresee obstacles and free space during navigation. More recently, 3D laser rangefinders and LiDARs are being developed, such as the sensor presented in [1], but this one is not compact enough for MAVs. Another disadvantage of laser-based technologies is their active sensing nature, which requires more power to operate and their measurements are more vulnerable to detection and corruption (e.g., due to dark/reflective surfaces) than vision-based solutions. Time-of-flight (ToF) cameras as well as red, green, blue plus depth (RGB-D) sensors like the Microsoft Kinect^®^ are also very popular for robot navigation. They have been adopted for low-sunlight conditions and mainly indoor navigation of MAVs [2] due to its structured infrared light projection and short range sensing (under 5 m) [3]. Hence, a lightweight imaging system capable of instantly providing a large field of view (FOV) with acceptable resolutions is essential for MAV applications in 3D space. These state-of-the-art sensors’ pitfalls motivate the design and analysis of our omnistereo sensor.

### 1.3. Related Work

Using omnidirectional images alone and motion—like the approaches taken in [4,5]—have been proposed to map and localize a robot. Omnidirectional vision using a single mirror for the flight of large UAVs was first attempted in [6]. In [7], Hrabar proposed the use of traditional horizontal stereo-based obstacle avoidance and path planing for AUVs, but these techniques were only tested in a scaled-down air vehicle simulator (AVS). Omnidirectional catadioptric cameras can be aided by structured light such as the prototypes presented in [8] and more flexible configurations demonstrated in [9]. Alternatively, stereo cameras can provide passive, instantaneous 3D information for robot mapping and navigation (including UAVs [10]). Intuitively, omnidirectional stereo (omnistereo) can be achieved through circular arrangements of multiple perspective cameras with overlapping views. Higher resolution panoramas can be achieved by rotating a linear camera as presented in [11], but this approach suffers from motion blur in dynamic environments. We point the reader to [12] for a detailed study of multiple view geometry, and [13] for a compendium of geometric computer vision concepts. Instead, our solution to omnistereo vision consists of a ‘catadioptric’ system by employing cameras and mirrors [14].

Throughout the years, [15,16,17,18,19,20] are some of the works that have applied various omnistereo catadioptric configurations for ground mobile robots. Unfortunately, these systems are not compact since they use separate camera-mirror pairs, which are known to experience synchronization issues. In [21], Yi and Ahuja described a configuration using a mirror and a concave lens for omnistereo, but it rendered a very short baseline in comparison to the two-mirror configurations. Originally, Nayar and Peri [22] studied 9 possible folded-catadioptric configurations for a single-camera omnistereo imaging system. Eventually, a catadioptric system using two hyperbolic mirrors in a vertical configuration was implemented by He *et al.* [23]. Their omnistereo sensor provides a lengthy baseline at the expense of a very tall system. In the past [24], we developed a novel omnistereo catadioptric rig consisting of a perspective camera coaxially-aligned with two spherical mirrors of distinct radii (in a “folded" configuration). One caveat of spherical mirrors is their non-centrality; they do not satisfy the single effective viewpoint (SVP) constraint (discussed in Section 2.2) but rather a locus of viewpoints is obtained [25].

### 1.4. Proposed Sensor

We design a SVP-compliant omnistereo system based on the folded, catadioptric configuration with hyperboloidal mirrors. Our approach resembles the work of Jang, Kim, and Kweon [26], who first implemented an omnistereo system using a pair of hyperbolic mirrors and a single camera. However, their sensor’s characteristics were not studied in order to justify their design parameters and capabilities, which we do in our case.

It is true that an omnidirectional catadioptric system sacrifices spatial resolution on the imaging sensor (analyzed in Section 3.4). However, our sensor offers practical advantages such as reduced cost, acceptable weight, and truly-instantaneous pixel-disparity correspondences since the same single camera-lens operates for both views, so mis-synchronization issues do not exist. In fact, we believe we are the first to present a single-camera catadioptric omnistereo solution for MAVs. The initial geometry of our model was proposed in [27]. Now, we perform an extensive analysis of our model’s parameters (Section 2) involving its geometric projection (Section 3) that are obtained as a constrained numerical optimization solution devising the sensor’s real-life application to MAVs passive range sensing (Section 4). We also show how the panoramic images are obtained, where we find correspondences and triangulate 3D points for which an uncertainty model is introduced (Section 5). Finally, we present our experimental results and evaluation for 3D sensing with the proposed omnistereo sensor (Section 6), and we discuss the future direction of our work in Section 7.

## 2. Sensor Design

**Figure 1:**
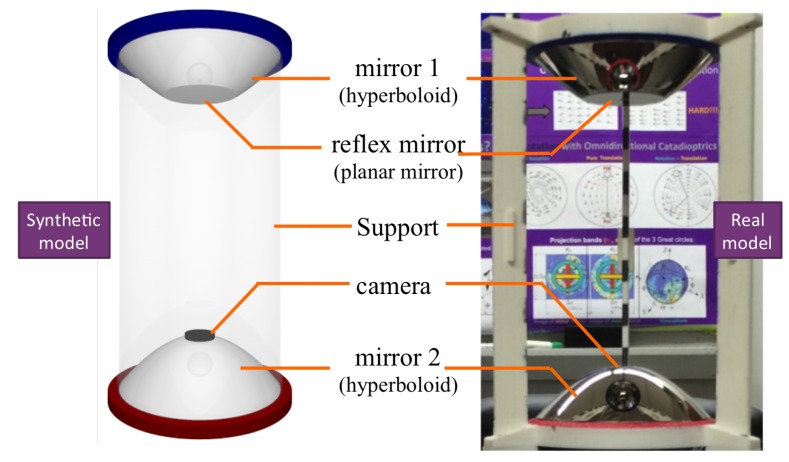
Synthetic and real prototypes for the catadioptric single-camera omnistereo system.

**Figure 2:**
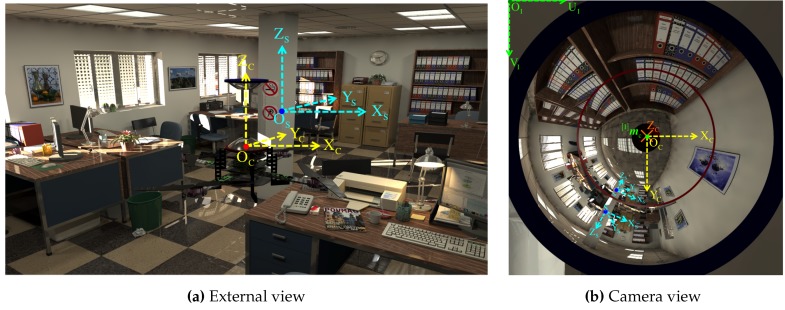
Photo-realistic synthetic scene: (**a**) Side-view of the quadrotor with the omnistereo rig in an office environment; (**b**) the image captured by the system’s camera using this pose.

Figure 1 shows the single-camera catadioptric omnistereo vision system that we specifically design to be mounted on top of our micro quadrotors (manufactured by Ascending Technologies [28]). It consists of (1) one hyperboloid-planar mirror at the top; (2) one hyperboloidal mirror at the bottom; and (3) a high-resolution USB camera also at the bottom (inside the bottom mirror and looking up). The components are housed and supported by a (4) transparent tube or plastic standoffs (for the real-life prototype shown in Figure 13). The choice of the hyperboloidal reflectors owes to three reasons: it is one of the four non-degenerated conic shapes satisfying the SVP constraint [29]; it allows a wider vertical FOV than elliptical and planar mirrors; and it does not require a telescopic (orthographic) lens for imaging as with paraboloidal mirrors (so our system can be downsized). In addition, the planar part of mirror 1 works as a reflex mirror, which in part reduces distortion caused by dual conic reflections. Based on the SVP property, the system obtains two radial images of the omnidirectional views in the form of an inner and an outer ring as illustrated in Figure 2a,b). Nevertheless, the unique set of parameters describing the entire system categorizes it as a “global camera model" given by [13] because changing the value of any parameter in the model affects the overall projection function of visible light rays in the scene as well as other computational imaging factors such as depth resolution and overlapping field of view, which we attempt to optimize with the following design subsections. Please, refer to Appendix A for clarification on our symbolic notation.

### 2.1. Model Parameters

**Figure 3:**
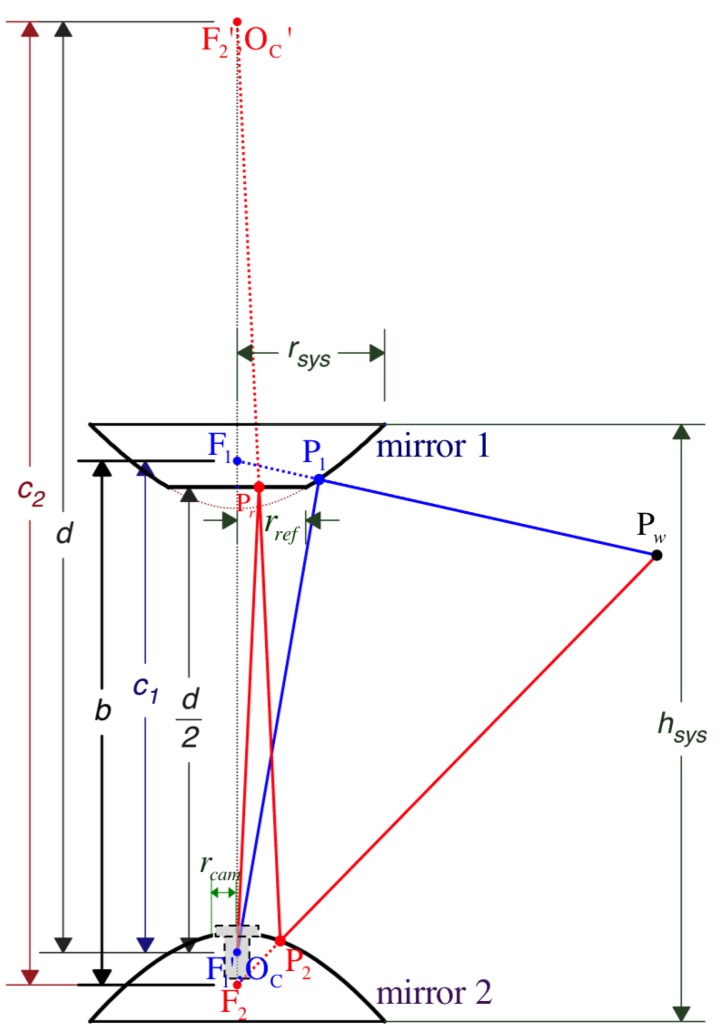
Geometric model and observable design parameters.

In the configuration of Figure 3, mirror 1’s real or primary focus is $F_{1}$, which is separated by a distance $c_{1}$ from its virtual or secondary focus, $F_{1}^{\prime }$, at the bottom. Without loss of generality, we make both the camera’s pinhole and $F_{1}^{\prime }$ coincide with the origin of the camera’s coordinate system, $O_{C}$. This way, the position of the primary focus, $F_{1}$, can be referenced by vector ${}^{\left[C\right]}\;f_{1}={\left[\begin{array}{ccc} 0, & 0, & c_{1} \end{array}\right]}^{T}$ in Cartesian coordinates with respect to the camera frame, $\left[C\right]$. Similarly, the distance between the foci of mirror 2, $F_{2}$ and $F_{2}^{\prime }$, is measured by $c_{2}$. Here, we use the planar (reflex) mirror of radius $r_{ref}$ and unit normal vector

(1) $$\begin{array}{c} {}^{\left[C\right]}\;{\hat{n}}_{ref}=[\begin{array}{ccc} 0, & 0, & -1 \end{array}] \end{array}$$

in order to project the real camera’s pinhole located at $O_{C}$ as a virtual camera $O_{C}^{\prime }$ coinciding with the virtual focal point $F_{2}^{\prime }$ positioned at ${}^{\left[C\right]}\;f_{2v}={\left[\begin{array}{ccc} 0, & 0, & d \end{array}\right]}^{T}$. We achieve this by setting d/2 as the symmetrical distance from the reflex mirror to $O_{C}$ and from the reflex mirror to $O_{C}^{\prime }$. With respect to $\left[C\right]$, mirror 2’s primary focus, $F_{2}$, results in position ${}^{\left[C\right]}\;f_{2}={\left[\begin{array}{ccc} 0, & 0, & d-c_{2} \end{array}\right]}^{T}$. It yields the following expression for the reflective plane:

(2) $$\begin{array}{c} {}^{\left[C\right]}\;{\hat{n}}_{ref}^{T}{}^{\left[C\right]}\;x=-d/2 \end{array}$$

The profile of each hyperboloid is determined by independent parameters $k_{1}$ and $k_{2}$, respectively. Their reflective vertical field of view (vFOV) are indicated by angles $\alpha_{1}$ and $\alpha_{2}$. They play an important role when designing the total vFOV of the system, $\alpha_{sys}$, formally defined by Equation (54) and illustrated in Figure 5. Also importantly, while performing stereo vision, it is to consider angle $\alpha_{SROI}$, which measures the common (overlapping) vFOV of the omnistereo system. The camera’s nominal field of view $\alpha_{cam}$ and its opening radius $r_{cam}$ also determine the physical areas of the mirrors that can be fully imaged. Theoretically, the mirrors’ vertical axis of symmetry (coaxial configuration) produces two image points that are radially collinear. This property is advantageous for the correspondence search during stereo sensing (Section 5) with a baseline measured as

(3) $$b=c_{1}+c_{2}-d$$

Among design parameters, we also include the total height of the system, $h_{sys}$, and weight $m_{sys}$, both being formulated in Section 2.3.

To summarize, the model has 6 primary design parameters given as a vector

(4) $$\begin{array}{c} \theta =\left[\begin{array}{cccccc} c_{1}, & c_{2}, & k_{1}, & k_{2}, & d, & r_{sys} \end{array}\right] \end{array}$$

in addition to by-product parameters such as

$$\begin{array}{c} \left[\begin{array}{cccccccccc} b, & h_{sys}, & r_{ref}, & r_{cam}, & m_{sys}, & \alpha_{1}, & \alpha_{2}, & \alpha_{sys}, & \alpha_{SROI}, & \alpha_{cam} \end{array}\right] \end{array}$$

In Section 4, we perform a numerical optimization of the parameters in **θ** with the goal to maximize the baseline, *b*, required for life-size navigational stereopsis. At the same time, we restrict the overall size of the rig (Section 2.3) without sacrificing sensing performance characteristics such as vertical field of view, spatial resolution, and depth resolution. In the upcoming subsections, we first derive the analytical solutions for the forward projection problem in our coaxial stereo configuration as a whole. In Section 3.2, we derive the back-projection equations for lifting 2D image points into 3D space.

### 2.2. Single Viewpoint (SVP) Configuration for OmniStereo

As a central catadioptric system, its projection geometry must obey the existence of the so-called single effective viewpoint (SVP). While the SVP guarantees that true perspective geometry can always be recovered from the original image, it limits the selection of mirror profiles to a set of conics. Generally, a circular hyperboloid of revolution (about its axis of symmetry) conforms to the SVP constraint as demonstrated by Baker and Nayar in [30]. Since a hyperboloidal mirror has two foci, the effective viewpoint is the primary focus F inside the physical mirror and the secondary (outer) focus $F^{\prime }$ is where the centre (pinhole) of the perspective camera should be placed for depicting a scene obeying the SVP configuration discussed in this section.

First of all, a hyperboloid *i* can be described by the following parametric equation:

(5) $$\begin{array}{c} \frac{{\left(z_{i}-z_{0_{i}}\right)}^{2}}{a_{i}^{2}}-\frac{r_{i}^{2}}{b_{i}^{2}}=1,\;\text{with}\;a_{i}=\frac{c_{i}}{2}\sqrt{\frac{k_{i}-2}{k_{i}}},\;b_{i}=\frac{c_{i}}{2}\sqrt{\frac{2}{k_{i}}} \end{array}$$

where $z_{0_{i}}=\frac{c_{i}}{2}$ is the offset (shift) position of the focus along the Z-axis from the origin $O_{C}$, and $r_{i}$ is the orthogonal distance to the axis of revolution / symmetry (i.e. the Z-axis) from a point $P_{i}$ on its surface.

In fact, the position of a valid point $P_{i}$ is constrained within the mirror’s physical surface of reflection, which is radially limited by $r_{i,min}$ and $r_{i,max}$, such that:

(6) $$\begin{array}{c} r_{i}=\sqrt{x_{i}^{2}+y_{i}^{2}},\;\;\text{for}\;\;r_{i,min}\leq r_{i}\leq r_{i,max},\;\forall i\in \left\{1,2\right\} \end{array}$$

and $r_{1,min}=r_{ref},\;r_{1,max}=r_{sys},\;r_{2,min}=r_{cam},\;r_{2,max}=r_{sys}$. Observe that the radius of the system is the upper bound for both mirrors (Figure 3). In addition, the hyperboloids profiled by Equation (5) must obey the following conical constraints:

(7) $$\begin{array}{c} \forall i\in \left\{1,2\right\}\left(c_{i}>0\wedge k_{i}>2\right) \end{array}$$

*k* is a constant parameter (unit-less) inversely related to the mirror’s curvature or more precisely, the eccentricity $\varepsilon_{c}$ of the conic. In fact, $\varepsilon_{c}>1$ for hyperbolas, yet a plane is produced when $\varepsilon_{c}\to \infty$ or k=2.

We devise $M_{i}$ as the set of all the reflection points $P_{i}$ with coordinates $\left(x_{i},y_{i},z_{i}\right)$ laying on the surface of the respective mirror *i* within bounds. Formally,

(8) $$\begin{array}{c} M_{i}:=\left\{P_{i}\in R^{3}\;|\;\frac{{\left(z_{i}-z_{0_{i}}\right)}^{2}}{a_{i}^{2}}-\frac{r_{i}^{2}}{b_{i}^{2}}=1\wedge \text{Equation}\;(6)\wedge \text{Equation}\;(7)\right\} \end{array}$$

In our model, we describe both hyperboloidal mirrors, 1 and 2, with respect to the camera frame $\left[C\right]$, which acts as the common origin of the coordinate system. Therefore,

(9) $$\begin{array}{cc} z_{0_{1}} & =\frac{c_{1}}{2} \end{array}$$

(10) $$\begin{array}{cc} z_{0_{2}} & =d-\frac{c_{2}}{2} \end{array}$$

By expanding Equation (5) with their respective index terms, it becomes

(11) $$\begin{array}{cc} {\left(z_{1}-\frac{c_{1}}{2}\right)}^{2}-r_{1}^{2}\left(\frac{k_{1}}{2}-1\right) & =\frac{c_{1}^{2}}{4}\left(\frac{k_{1}-2}{k_{1}}\right) \end{array}$$

(12) $$\begin{array}{cc} {\left(z_{2}-d+\frac{c_{2}}{2}\right)}^{2}-r_{2}^{2}\left(\frac{k_{2}}{2}-1\right) & =\frac{c_{2}^{2}}{4}\left(\frac{k_{2}-2}{k_{2}}\right) \end{array}$$

Additionally, we define the function $f_{z_{i}}:r\mapsto z_{i}$ to find the corresponding $z_{i}$ component from a given *r* value as

(13) $$\begin{array}{c} \begin{array}{cc} f_{z_{i}}\left(r\right):= & \left\{\begin{array}{cc} z_{0_{i}}+\gamma_{i} & \text{if}\;i=1\wedge \;\text{Equation}\;(6)\\ z_{0_{i}}-\gamma_{i} & \text{if}\;i=2\wedge \;\text{Equation}\;(6)\\ None & \text{otherwise} \end{array}\right. \end{array} \end{array}$$

where $\gamma_{i}=\frac{a_{i}}{b_{i}}\sqrt{b_{i}^{2}+r_{i}^{2}}$.

The inverse relation $f_{r_{i}}:z\mapsto \left\{+r_{i},-r_{i}\right\}$ can be also implemented as

(14) $$\begin{array}{c} \begin{array}{cc} f_{r_{i}}\left(z\right):= & \left\{\begin{array}{cc} \pm b_{i}\Gamma_{i} & \text{if}\;i\in \{1,2\}\wedge \;\text{Equation}\;(6)\\ None & \text{otherwise} \end{array}\right. \end{array} \end{array}$$

where $\Gamma_{i}=\sqrt{\frac{{\left(z-z_{0_{i}}\right)}^{2}}{a_{i}^{2}}-1}$, so a valid input *z* can be associated with both positive and negative solutions $r_{i}$.

### 2.3. Rig Size

In the attempt to evaluate the overall system size, we consider the height and weight variables due to the primary design parameters, **θ**.

First, the height of the system, $h_{sys}$ can be estimated from the functional relationships $f_{z_{1}}$ and $f_{z_{2}}$ defined in Equation (13), which can provide the respective z−component values at the out-most point on the mirror’s surface. More specifically, knowing $r_{sys}$, we get

(15) $$\begin{array}{c} h_{sys}=z_{max}-z_{min} \end{array}$$

where $z_{max}=f_{z_{1}}\left(r_{sys}\right)$ and $z_{min}=f_{z_{2}}\left(r_{sys}\right)$.

The rig’s weight can be indicated by the total resulting mass of the main “tangible” components:

(16) $$\begin{array}{c} m_{sys}=m_{cam}+m_{tub}+m_{mir} \end{array}$$

where the mass of the camera-lens combination is $m_{cam}$; the mass of the support tube $m_{tub}$ can be estimated from its cylindrical volume $V_{tub}$ and material density $\rho_{tub}$, and the mass due to the mirrors

(17) $$\begin{array}{c} \begin{array}{cc} m_{mir} & =V_{mir}\rho_{mir}\\ & =\left(V_{1}+V_{ref}+V_{2}\right)\rho_{mir} \end{array} \end{array}$$

For computing the volume of the hyperboloidal shell, $V_{i}$ for mirror *i*, we apply a “ring method” of volume integration. By assuming all mirror material has the same wall thickness $\tau_{m}$, we acquire $V_{i}$ by integrating the horizontal cross-sections area along the Z-axis. Each ring area depends on its outer and inner circumferences that vary according to radius $r{|}_{z}$ for a given height *z*. Equation (14) establishes the functional relation ${r_{i}}^{+}=f_{r_{i}}\left(z\right)$, from which we only need its positive answer. We let *A* be the function that computes the ring area of constant thickness $\tau_{m}$ for a variable outer radius $r_{i}$

(18) $$\begin{array}{c} \begin{array}{cc} A(r_{i}) & =\pi r_{i}^{2}-\pi {\left(r_{i}-\tau_{m}\right)}^{2}\\ & =\pi \tau_{m}\left(2r_{i}-\tau_{m}\right) \end{array} \end{array}$$

We consider the definite integral evaluated in the *z* interval bounded by its height limits, which are correlated with its radial limits Equation (6) and can be obtained via the $f_{z_{i}}$ defined in Equation (13), such that

(19) $$\begin{array}{c} z_{i,min}=f_{z_{i}}\left(r_{i,min}\right)\;\;\text{and}\;\;z_{i,max}=f_{z_{i}}\left(r_{i,max}\right) \end{array}$$

Then, we proceed to integrate Equation (18), so the shell volume for each hyperboloidal mirror is defined as

(20) $$\begin{array}{cc} V_{i} & =\int_{z_{i,min}}^{z_{i,max}}A\left(r_{i}\right)\mathrm{dz} \end{array}$$

Finally, since the reflex mirror piece is just a solid cylinder of thickness $\tau_{m}$, its volume is simply

(21) $$\begin{array}{c} V_{ref}=\tau_{m}\pi r_{ref}^{2} \end{array}$$

## 3. Projective Geometry

### 3.1. Analytical Solutions to Projection (Forward)

Assuming a central catadioptric configuration for the mirrors and camera system (Section 2.2), we derive the closed-form solution to the imaging process (forward projection) for an observable point $P_{w}$, positioned in three-dimensional Euclidean space, $R^{3}$, with respect to the reference frame, $\left[C\right]$, as vector ${}^{\left[C\right]}\;p_{w}={\left[\begin{array}{ccc} x_{w}, & y_{w}, & z_{w} \end{array}\right]}^{T}$. In addition, we assume all reference frames such as $\left[F_{1}\right]$ and $\left[F_{2}\right]$ have the same orientation as $\left[C\right]$.

For mathematical stability, we must constrain that all projecting world points lie outside the mirror’s volume:

(22) $$\begin{array}{c} f_{r_{i}}\left(z_{w}\right)<\rho_{w},\;\text{where}\;\rho_{w}=\sqrt{x_{w}^{2}+y_{w}^{2}} \end{array}$$

where $f_{r_{i}}$ is defined by Equation (14) and $\rho_{w}$ measures the horizontal range to $P_{w}$.

**Figure 4:**
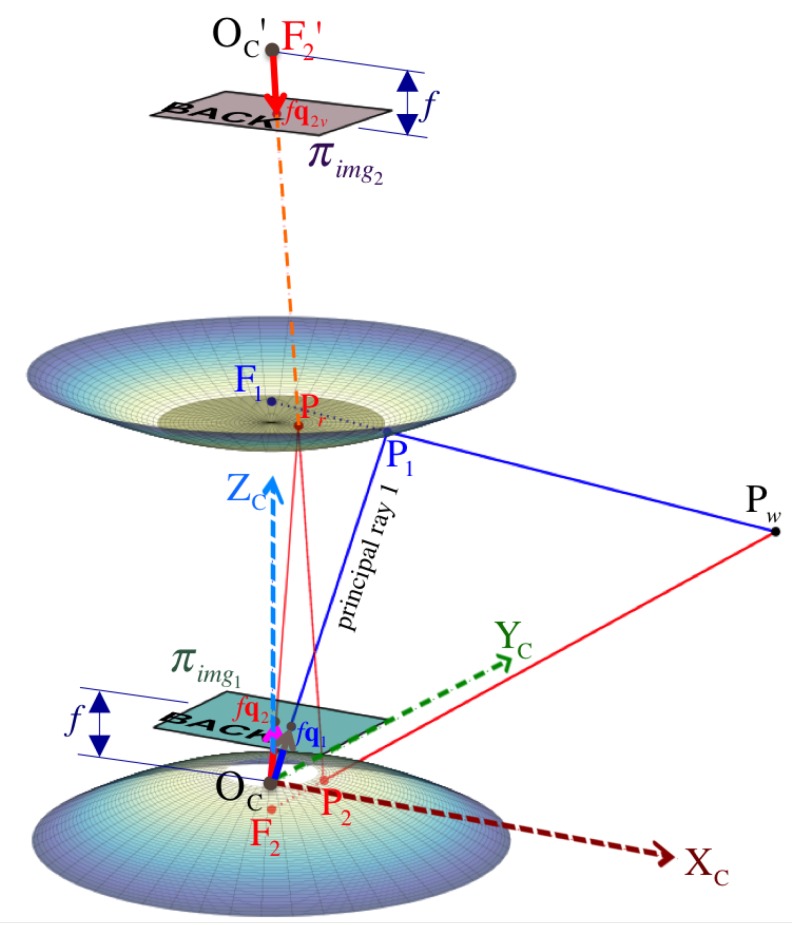
Omnistereo projection of a 3D point $P_{w}$ to obtain image points ${}^{\left[I\right]}\;m_{1}$ and ${}^{\left[I\right]}\;m_{2}$.

$P_{w}$ is imaged at pixel position ${}^{\left[I\right]}\;m_{1}$ after its reflection as point $P_{1}$ on the hyperboloidal surface of mirror 1 (Figure 4). On the other hand, the second image point’s position, ${}^{\left[C\right]}\;m_{2}$, due to reflection point $P_{2}$ on mirror 2 is rather obtained indirectly after an additional point $P_{r}$ is reflected at ${}^{\left[C\right]}\;p_{ref}$ on the reflex mirror represented via Equation (32).

First, for $P_{w}$’s reflection point via mirror 1 at position vector ${}^{\left[C\right]}\;p_{1}$, we use $\lambda_{1}$ as the parametrization term for the line equation passing through $F_{1}$ toward $P_{w}$ with direction ${}^{\left[F_{1}\right]}\;d_{1}={}^{\left[C\right]}\;p_{w}-{}^{\left[C\right]}\;f_{1}$. The position of any point $P_{1}$ on this line is given by:

(23) $${}^{\left[C\right]}\;p_{1}={}^{\left[C\right]}\;f_{1}+\lambda_{1}{}^{\left[F_{1}\right]}\;d_{1}$$

Substituting Equation (23) into Equation (11), we obtain:

$$\begin{array}{cc} {\left(\lambda_{1}\left(z_{w}-c_{1}\right)+\frac{c_{1}}{2}\right)}^{2}-\left(\lambda_{1}^{2}x_{w}^{2}+\lambda_{1}^{2}y_{w}^{2}\right)\left(\frac{k_{1}}{2}-1\right) & \\ -\frac{c_{1}^{2}}{4}\left(\frac{k_{1}-2}{k_{1}}\right) & =0 \end{array}$$

in order to solve for $\lambda_{1}$, which turns out to be

(24) $$\lambda_{1}=\frac{c_{1}}{\left\|{}^{\left[F_{1}\right]}\;d_{1}\right\|\sqrt{k_{1}\;\cdot \;\left(k_{1}-2\right)}-k_{1}\left(z_{w}-c_{1}\right)}$$

where $\left\|{}^{\left[F_{1}\right]}\;d_{1}\right\|=\sqrt{x_{w}^{2}+y_{w}^{2}+{\left(z_{w}-c_{1}\right)}^{2}}$ is the Euclidean norm between $P_{w}$ and mirror 1’s focus, $F_{1}$.

In practice, we represent the reflection point’s position ${}^{\left[C\right]}\;p_{1}$ as a matrix-vector multiplication between the 3×4 transformation matrix $K_{1}=\left[\begin{array}{cc} \lambda_{1}I_{\left(3\right)}, & \left(1-\lambda_{1}\right){}^{\left[C\right]}\;f_{1} \end{array}\right]$ and the point’s position vector ${}^{\left[C\right]}\;p_{w,h}={\left[\begin{array}{cccc} x_{w}, & y_{w}, & z_{w}, & 1 \end{array}\right]}^{T}$ in homogeneous coordinates:

(25) $${}^{\left[C\right]}\;p_{1}=K_{1}{}^{\left[C\right]}\;p_{w,h}$$

Note that ${}^{\left[C\right]}\;p_{1}$’s elevation angle, $\theta_{1}$, must be bounded as

(26) $$\begin{array}{c} \theta_{1,min}\leq \theta_{1}\leq \theta_{1,max} \end{array}$$

where $\theta_{1,min}$ and $\theta_{1,max}$ are the angular elevation limits for the real reflective area of the hyperboloid.

Finally, the reflection point $P_{1}$ with position ${}^{\left[C\right]}\;p_{1}$ can now be perspectively projected as a pixel point located at ${}^{\left[I\right]}\;m_{1}={\left[\begin{array}{cc} u_{1}, & v_{1} \end{array}\right]}^{T}$ on the image. In fact, the entire imaging process of $P_{w}$ via mirror 1 can be expressed in homogeneous coordinates as:

(27) $${}^{\left[I\right]}\;m_{1,h}=\zeta_{1}K_{c}K_{1}{}^{\left[C\right]}\;p_{w,h}$$

where the scalar $\zeta_{1}=1/z_{1}=1/\left(c_{1}+\lambda_{1}\left(z_{w}-c_{1}\right)\right)$ is the perspective normalizer that maps the *principal ray* passing through $p_{1}$ onto a point ${}^{\left[C\right]}\;q_{1}={\left[\begin{array}{ccc} x_{q_{1}}, & y_{q_{1}}, & 1 \end{array}\right]}^{T}$ on the normalized projection plane ${\hat{\pi }}_{img_{1}}$. The traditional 3×3 intrinsic matrix of the camera’s pinhole model is

(28) $$\begin{array}{c} K_{c}=\left[\begin{array}{ccc} f_{u} & s & u_{c}\\ 0 & f_{v} & v_{c}\\ 0 & 0 & 1 \end{array}\right] \end{array}$$

in which $f_{u}=f/h_{x}$ and $f_{v}=f/h_{y}$ are based on the focal length *f* and the pixel dimension $\left(h_{x},h_{y}\right)$, *s* is the skew parameter, and ${}^{\left[I\right]}\;m_{c}={\left[\begin{array}{cc} u_{c}, & v_{c} \end{array}\right]}^{T}$ is the optical center position on the image $\left[I\right]$. Figure 4 illustrates the projection point $f{}^{\left[C\right]}\;q_{1}$ on the respective image plane $\pi_{img_{1}}$.

Similarly, we provide the analytical solution for the forward projection of $P_{w}$ via mirror 2 by first considering the position of reflection point $P_{2}$:

(29) $${}^{\left[C\right]}\;p_{2}=K_{2}{}^{\left[C\right]}\;p_{w,h}$$

where $K_{2}=\left[\begin{array}{cc} \lambda_{2}I_{\left(3\right)}, & \left(1-\lambda_{2}\right){}^{\left[C\right]}\;f_{2} \end{array}\right]$ is similar to the transformation matrix $K_{1}$, but obviously it now uses ${}^{\left[C\right]}\;f_{2}$ and

(30) $$\lambda_{2}=\frac{c_{2}}{\left\|{}^{\left[F_{2}\right]}\;d_{2}\right\|\sqrt{k_{2}\;\cdot \;\left(k_{2}-2\right)}+k_{2}\left(z_{w}-\left(d-c_{2}\right)\right)}$$

with direction vector’s norm

(31) $$\begin{array}{c} \left\|{}^{\left[F_{2}\right]}\;d_{2}\right\|=\left\|{}^{\left[C\right]}\;p_{w}-{}^{\left[C\right]}\;f_{2}\right\|=\sqrt{x_{w}^{2}+y_{w}^{2}+{\left(z_{w}-\left(d-c_{2}\right)\right)}^{2}} \end{array}$$

For completeness, note that the physical projection via mirror 2 is incident to the reflex mirror at

(32) $$\begin{array}{c} {}^{\left[C\right]}\;p_{ref}={}^{\left[C\right]}\;f_{2v}+\lambda_{ref}\left({}^{\left[C\right]}\;p_{2}-{}^{\left[C\right]}\;f_{2v}\right) \end{array}$$

where $\lambda_{ref}=\frac{d}{2(d-z_{2})}$ according to Equation (2) in the theoretical model. Ultimately, ignoring any astigmatism and chromatic aberrations introduced by the reflex mirror, and because the same (and only) real camera with $K_{c}$ is used for imaging, we obtain the projected pixel position ${}^{\left[I\right]}\;m_{2,h}={\left[\begin{array}{ccc} u_{2}, & v_{2}, & 1 \end{array}\right]}^{T}$:

(33) $${}^{\left[I\right]}\;m_{2,h}=\zeta_{2}K_{c}K_{ref}K_{2}{}^{\left[C\right]}\;p_{w,h}$$

where $\zeta_{2}=1/\left(d-z_{2}\right)$ is the perspective normalizer to find ${}^{\left[C\right]}\;q_{2}$ on the normalized projection plane, ${\hat{\pi }}_{img_{2}}$.

Due to planar mirroring via the reflex mirror, ${}_{\left[C\right]}^{\left[C^{\prime }\right]}\;K_{ref}$ is used to change the coordinates of $P_{2}$ from $\left[C\right]$ onto the virtual camera frame, $\left[C^{\prime }\right]$, located at ${}^{\left[C\right]}\;f_{2v}$. Hence,

(34) $$\begin{array}{c} {}_{\left[C\right]}^{\left[C^{\prime }\right]}\;K_{ref}=\left[\begin{array}{ccc} I_{\left(3\right)}+2D_{{\hat{n}}_{ref}}, & & {}^{\left[C\right]}\;f_{2v} \end{array}\right] \end{array}$$

where the 3×1 unit normal vector of the reflex mirror plane, ${}^{\left[C\right]}\;{\hat{n}}_{ref}$ given in Equation (1), is mapped into its corresponding 3×3 diagonal matrix $D_{{\hat{n}}_{ref}}$, via the relationship:

(35) $$\begin{array}{c} D_{{\hat{n}}_{ref}}\leftarrow I_{\left(3\right)}\mathrm{diag}\;\left({}^{\left[C\right]}\;{\hat{n}}_{ref}\right) \end{array}$$

**Figure 5:**
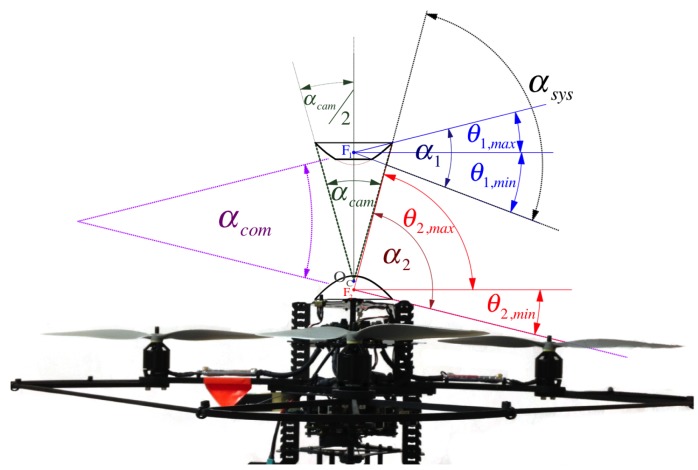
Vertical Field of View (vFOV) angles: $\alpha_{1}$ and $\alpha_{2}$ are the individual angles of the mirrors formed by their respective elevation limits $\theta_{1/2,min/max}$; $\alpha_{sys}$ is the overall vFOV angle of the system; and $\alpha_{SROI}$ measures the overlapping region conceived between $\alpha_{1}$ and $\alpha_{2}$.

It is convenient to define the forward projection functions $f_{\varphi_{1}}\left({}^{\left[C\right]}\;p\right)$ and $f_{\varphi_{2}}\left({}^{\left[C\right]}\;p\right)$ for a 3D point P whose position vector is known with respect to $\left[C\right]$ and which is situated within the vertical field of view $\alpha_{i}$ of mirror *i* (for i∈{1,2}) indicated in Figure 5. Function $f_{\varphi_{i}}\left({}^{\left[C\right]}\;p\right)$ maps ${}^{\left[C\right]}\;p$ to image point ${}^{\left[I\right]}\;m_{i}$ on frame $\left[I\right]$, such that $f_{\varphi_{i}}:R^{3}\mapsto R^{2}$,

(36) $$\begin{array}{cc} f_{\varphi_{i}}\left({}^{\left[C\right]}\;p\right):= & \left\{\begin{array}{cc} {}^{\left[C\right]}\;p\overset{\text{Equation}\;(27)}{\mapsto }{}^{\left[I\right]}\;m_{1} & \;\text{if}\;i=1\wedge \;\text{Equations}\;(37)\;\text{and}\;(22)\\ {}^{\left[C\right]}\;p\overset{\text{Equation}\;(33)}{\mapsto }{}^{\left[I\right]}\;m_{1} & \;\text{if}\;i=2\wedge \;\text{Equations}\;(37)\;\text{and}\;(22)\\ None & \;\text{otherwise} \end{array}\right. \end{array}$$

In fact, ${}^{\left[I\right]}\;m_{i}$ is considered valid if it is located within the imaged radial bounds, such that:

(37) $$\begin{array}{c} \;{}^{\left[I_{C_{i}}\right]}\;\|{}^{\left[I_{i}\right]}\;m_{r_{i,min}}\|\;\leq \;{}^{\left[I_{C_{i}}\right]}\;{}^{\left[I\right]}\;m_{i}\;\leq \;{}^{\left[I_{C_{i}}\right]}\;\|{}^{\left[I\right]}\;m_{r_{i,max}}\| \end{array}$$

where the frame of reference $\left[I_{C_{i}}\right]$ implies that its origin is the image center ${}^{\left[I\right]}\;m_{c}={\left[\begin{array}{cc} u_{c_{i}}, & v_{c_{i}} \end{array}\right]}^{T}$ of the $\left[I_{i}\right]$ masked image (Figure 7). Therefore, the magnitude (norm) of any position ${}^{\left[I_{C_{i}}\right]}\;m$ in pixel space $\left[I_{C_{i}}\right]$ can be measured as

(38) $$\begin{array}{c} {}^{\left[I_{C_{i}}\right]}\;\|{}^{\left[I_{i}\right]}\;m\|:=\;\|{}^{\left[I_{i}\right]}\;m-{}^{\left[I_{i}\right]}\;m_{c}\|=\sqrt{{\left(u-u_{c}\right)}^{2}+{\left(v-v_{c}\right)}^{2}} \end{array}$$

In particular, ${}^{\left[I_{C_{i}}\right]}\|\;{}^{\left[I\right]}\;m_{r_{i,lim}}$ is the image radius obtained from the projection ${}^{\left[I\right]}\;m_{r_{i,lim}}\leftarrow f_{\varphi_{i}}\left({}^{\left[C\right]}\;p_{i,lim}\right)$ corresponding to a particular point coincident with the line of sight of the radial limit $r_{i,lim}$—it being either $r_{sys}$, $r_{ref}$, or $r_{cam}$ as indicated by Equation (6).

### 3.2. Analytical Solutions to Back Projection

The back projection procedure establishes the relationship between the 2D position of a pixel point ${}^{\left[I\right]}\;m_{i}={\left[\begin{array}{cc} u, & v \end{array}\right]}^{T}$ on the image $\left[I_{i}\right]$ and its corresponding 3D projective direction vector $v_{i}$ toward the observed point $P_{w}$ in the world.

Initially, the pixel point ${}^{\left[I\right]}\;m_{1}$ (imaged via mirror 1) is mapped as $Q_{1}$ onto the normalized projection plane ${\hat{\pi }}_{img_{1}}$ with coordinates ${}^{\left[C\right]}\;q_{1}={\left[\begin{array}{ccc} x_{q_{1}}, & y_{q_{1}}, & 1 \end{array}\right]}^{T}$ by applying the inverse transformation of the camera intrinsic matrix Equation (28) as follows:

(39) $${}^{\left[C\right]}\;q_{1}={}_{\left[C\right]}^{\left[I\right]}\;K_{c}^{-1}{}^{\left[I\right]}\;m_{1,h}=\left[\begin{array}{ccccc} \frac{1}{f_{u}} & & -\frac{s}{f_{u}f_{v}} & & \frac{sv_{c}-f_{v}u_{c}}{f_{u}f_{v}}\\ 0 & & \frac{1}{f_{v}} & & -\frac{v_{c}}{f_{v}}\\ 0 & & 0 & & 1 \end{array}\right]\left[\begin{array}{c} u_{1}\\ v_{1}\\ 1 \end{array}\right]$$

For simplicity, we assume no distortion parameters exist, so we can proceed with the lifting step along the principal ray that passes through three points: the camera’s pinhole $O_{C}$, point $Q_{1}$ on the projection plane, and the reflection point $P_{1}$ (Figure 4). The vector form of this line equation can be written as:

(40) $$\begin{array}{cc} {}^{\left[C\right]}\;p_{1} & ={}^{\left[C\right]}\;o_{c}+t_{1}\left({}^{\left[C\right]}\;q_{1}-{}^{\left[C\right]}\;o_{c}\right)=t_{1}{}^{\left[C\right]}\;q_{1} \end{array}$$

By substituting Equation (40) into Equation (11), we solve for the parameter $t_{1}$, to get

(41) $$t_{1}=\frac{c_{1}}{k_{1}-\left\|{}^{\left[C\right]}\;q_{1}\right\|\sqrt{k_{1}\;\cdot \;\left(k_{1}-2\right)}}$$

where $\left\|{}^{\left[C\right]}\;q_{1}\right\|=\sqrt{x_{q_{1}}^{2}+y_{q_{1}}^{2}+1}$ is the distance between $Q_{1}$ and $O_{C}$.

Given ${}^{\left[F_{1}\right]}\;v_{1}$ as the direction vector leaving focal point $F_{1}$ toward the world point ${}^{\left[C\right]}\;P_{w}$. Through frame transformation ${}_{\left[C\right]}^{\left[F_{1}\right]}\;T_{1}{}^{\left[C\right]}\;p_{1,h}$, we get

(42) $$\begin{array}{c} {}^{\left[F_{1}\right]}\;v_{1}={}_{\left[C\right]}^{\left[F_{1}\right]}\;T_{1}{}^{\left[C\right]}\;p_{1,h}\;,\;\text{where}\;{{}_{\left[C\right]}^{\left[F_{1}\right]}\;T_{1}}_{\left(3\times 4\right)}=\left[\begin{array}{ccc} I_{\left(3\right)}, & & -{}^{\left[C\right]}\;f_{1} \end{array}\right] \end{array}$$

for ${}^{\left[C\right]}\;p_{1,h}$ as the homogeneous form of Equation (40). In fact, ${}^{\left[F_{1}\right]}\;v_{1}$ provides the back-projected angles (elevation $\theta_{1}$, azimuth $\psi_{1}$) from focus $F_{1}$ toward ${}^{\left[C\right]}\;P_{w}$:

(43) $$\begin{array}{cc} {}^{\left[F_{1}\right]}\;\theta_{1} & =\arcsin\;\left(\frac{z_{v_{1}}}{\left\|{}^{\left[F_{1}\right]}\;v_{1}\right\|}\right)=\arcsin\;\left(\frac{z_{1}-c_{1}}{\left\|{}^{\left[F_{1}\right]}\;v_{1}\right\|}\right) \end{array}$$

(44) $$\begin{array}{cc} {}^{\left[F_{1}\right]}\;\psi_{1} & =\arctan\;\left(\frac{y_{v_{1}}}{x_{v_{1}}}\right)=\arctan\;\left(\frac{y_{1}}{x_{1}}\right) \end{array}$$

where $\left\|{}^{\left[F_{1}\right]}\;v_{1}\right\|$ is the norm of the back-projection vector up to the mirror surface.

Using the same approach, we lift a pixel point ${}^{\left[I\right]}\;m_{2}$ imaged via mirror 2. Because the virtual camera $O_{C}^{\prime }$ located at ${}^{\left[C\right]}\;f_{2}={\left[\begin{array}{ccc} 0, & 0, & d-c_{2} \end{array}\right]}^{T}$ uses the same intrinsic matrix $K_{c}$, we can safely back-project pixel ${}^{\left[I\right]}\;m_{2}$ to $Q_{2v}$ on the normalized projection plane ${\hat{\pi }}_{img_{2}}$ as follows:

(45) $$\begin{array}{c} {}^{\left[C^{\prime }\right]}\;q_{2v}={}^{\left[C\right]}\;q_{2}=K_{c}^{-1}{}^{\left[I\right]}\;m_{2,h} \end{array}$$

where the inverse transformation of the camera intrinsic matrix $K_{c}^{-1}$ is given by Equation (28). Since the reflection matrix $K_{ref}$ defined in Equation (34) is bidirectional due to the symmetric position of the reflex mirror about $\left[C\right]$ and $\left[C^{\prime }\right]$, we can find the desired position of ${}^{\left[C\right]}\;q_{2v}$ with respect to $\left[C\right]$:

(46) $${}^{\left[C\right]}\;q_{2v}={}_{\left[C^{\prime }\right]}^{\left[C\right]}\;K_{ref}{}^{\left[C^{\prime }\right]}\;q_{2v,h}$$

which is equivalent to ${}^{\left[C\right]}\;q_{2v}={\left[\begin{array}{ccc} x_{q_{2v}}, & y_{q_{2v}}, & d-1 \end{array}\right]}^{T}$.

In Figure 4, we can see the principal ray that passes through the virtual camera’s pinhole $O_{C}^{\prime }$ and the reflection point $P_{2}$, so this line equation can be written as:

(47) $$\begin{array}{cc} {}^{\left[C\right]}\;p_{2} & ={}^{\left[C\right]}\;f_{2v}+t_{2}\left({}^{\left[C\right]}\;q_{2v}-{}^{\left[C\right]}\;f_{2v}\right) \end{array}$$

Solving for $t_{2}$ Equations (47) and (12), we get

(48) $$t_{2}=\frac{c_{2}}{k_{2}-\left\|{}^{\left[C\right]}\;q_{2}\right\|\sqrt{k_{2}\;\cdot \;\left(k_{2}-2\right)}}$$

where $\left\|{}^{\left[C\right]}\;q_{2}\right\|=\sqrt{x_{q_{2}}^{2}+y_{q_{2}}^{2}+1}$ is the distance between the normalized projection point $Q_{2}$ and the camera $O_{C}$ while considering Equation (46). Beware that the newly found location of $P_{2}$ is given with respect to the real camera frame, $\left[C\right]$.

Again, we obtain the back-projection ray

(49) $$\begin{array}{c} {}^{\left[F_{2}\right]}\;v_{2}={}_{\left[C\right]}^{\left[F_{2}\right]}\;T_{2}{}^{\left[C\right]}\;p_{2,h}\;,\;\text{where}\;{{}_{\left[C\right]}^{\left[F_{2}\right]}\;T_{2}}_{\left(3\times 4\right)}=\left[\begin{array}{ccc} I_{\left(3\right)}, & & -{}_{}^{}\;f_{2} \end{array}\right] \end{array}$$

in order to indicate the direction leaving from the primary focus $F_{2}$ toward $P_{w}$ through $P_{2}$. Here, the corresponding elevation and azimuth angles are respectively given by

(50) $$\begin{array}{cc} {}^{\left[F_{2}\right]}\;\theta_{2} & =\arcsin\;\left(\frac{z_{v_{2}}}{\left\|{}^{\left[F_{2}\right]}\;v_{2}\right\|}\right)=\arcsin\;\left(\frac{d-t_{2}}{\left\|{}^{\left[F_{2}\right]}\;v_{2}\right\|}\right) \end{array}$$

(51) $$\begin{array}{cc} {}^{\left[F_{2}\right]}\;\psi_{2} & =\arctan\;\left(\frac{y_{v_{2}}}{x_{v_{2}}}\right)=\arctan\;\left(\frac{y_{2}}{x_{2}}\right) \end{array}$$

where $\left\|{}^{\left[F_{2}\right]}\;v_{2}\right\|=\sqrt{x_{2}^{2}+y_{2}^{2}+{\left(c_{2}-t_{2}\right)}^{2}}$ is the magnitude of the direction vector from its reflection point $P_{2}$.

Like done for the (forward) projection, it is convenient to define the back-projection functions $f_{\beta_{1}}$ and $f_{\beta_{2}}$ for lifting a 2D pixel point ${}^{\left[I\right]}\;m$ within radial bounds validated by Equation (37) to their angular components ${}^{\left[F_{i}\right]}\;\left(\theta_{i},\psi_{i}\right)$ with respect to the respective foci frame $\left[F_{i}\right]$ (oriented like $\left[C\right]$) as indicated by Equations (43), (44), (50) and (51), such that $f_{\beta_{i}}:R^{2}\mapsto R^{2}$,

(52) $$\begin{array}{ccc} f_{\beta_{i}}\left({}^{\left[I\right]}\;m\right) & := & \left\{\begin{array}{cc} \left({}^{\left[I\right]}\;m\overset{\text{Equation}\;(43)}{\mapsto }{}^{\left[F_{1}\right]}\;\theta_{1},{}^{\left[I\right]}\;m\overset{\text{Equation}\;(44)}{\mapsto }{}^{\left[F_{1}\right]}\;\psi_{1}\right) & \text{if}\;i=1\\ \left({}^{\left[I\right]}\;m\overset{\text{Equation}\;(50)}{\mapsto }{}^{\left[F_{2}\right]}\;\theta_{2},{}^{\left[I\right]}\;m\overset{\text{Equation}\;(51)}{\mapsto }{}^{\left[F_{2}\right]}\;\psi_{2}\right) & \text{if}\;i=2\\ None & \neg \text{Equation}\;(37). \end{array}\right. \end{array}$$

### 3.3. Field-of-View

The horizontal FOV is clearly 360° for both mirrors. In other words, azimuths *ψ* can be measured in the interval $\left[0,2\pi \right)$ rad. As discussed previously, there exists a positive correlation between the vertical field of view (vFOV) angle $\alpha_{i}$ of mirror *i* and its profile parameter $k_{i}$, such that $\alpha_{i}\to 180{}^{\circ}$ as $k_{i}\to \infty$ (see Figure 9). As demonstrated in Figure 5, $\alpha_{i}$ is physically bounded by its corresponding elevation angles: $\theta_{i,max}$, $\theta_{i,min}$. Both vFOV angles, $\alpha_{1}$ and $\alpha_{2}$, are computed from their elevation limits as follows:

(53a) $$\begin{array}{cc} \alpha_{1} & =\theta_{1,max}-\theta_{1,min} \end{array}$$

(53b) $$\begin{array}{cc} \alpha_{2} & =\theta_{2,max}-\theta_{2,min} \end{array}$$

The overall vFOV of the system is also given from these elevation limits:

(54) $$\alpha_{sys}=\max\left(\theta_{1,max},\theta_{2,max}\right)-\min\left(\theta_{1,min},\theta_{2,min}\right)$$

**Figure 6:**
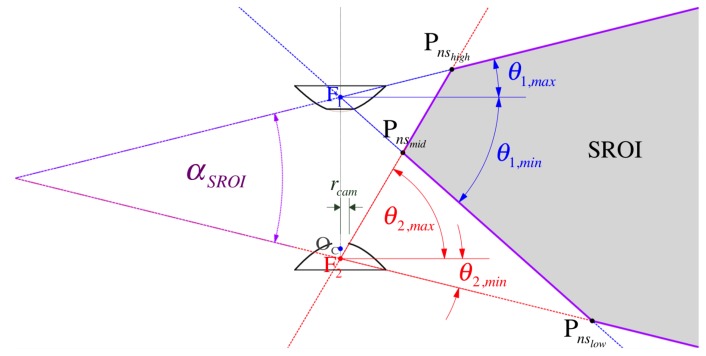
A cross section of the SROI (shaded area) formed by the intersection of view rays for the limiting elevations $\theta_{1/2,min/max}$. The nearest stereo (ns) points are labeled $P_{ns_{high}}$, $P_{ns_{mid}}$ and $P_{ns_{low}}$ since they are the vertices of the hull that near-bounds the set of usable points for depth computation from triangulation (Section 5.2). See Table 3 for the proposed sensor’s values.

Figure 6 highlights the the so-called common vFOV angle, $\alpha_{SROI}$, for the stereo region of interest (SROI) where the same point can be seen from both mirrors so point correspondences can be found (Section 5). In our model, $\alpha_{SROI}$ can be decided from the value of the three prevailing elevation angles ($\theta_{1,max}$, $\theta_{1,min}$, and $\theta_{2,min}$), such that:

(55) $$\alpha_{SROI}=\theta_{SROI,max}-\theta_{SROI,min}$$

where generally,

(56a) $$\begin{array}{cc} \theta_{SROI,min} & =\max(\theta_{1,min},\theta_{2,min}) \end{array}$$

(56b) $$\begin{array}{cc} \theta_{SROI,max} & =\min(\theta_{1,max},\theta_{2,max}) \end{array}$$

The shaded area in Figure 6 illustrates the SROI that is far-bounded by the set of triangulated points found at the maximum range due to minimum disparity $\Delta m_{12}=1\;\mathrm{px}$ in the discrete case (refer to Figure 17), such that

(57) $$\begin{array}{c} \begin{array}{cc} P_{fs}=\left\{\right.P_{w}\leftarrow f_{\Delta }\left(\left(\theta_{1},\psi_{1}\right),\left(\theta_{2},\psi_{2}\right)\right) & |(\theta_{1},\psi_{1})\leftarrow f_{\beta_{1}}\left(m_{1}\right)\\ & \wedge (\theta_{2},\psi_{2})\leftarrow f_{\beta_{2}}\left(m_{2}\right)\\ & \wedge \left.\Delta m_{12}=1,\mathrm{px}\right\} \end{array} \end{array}$$

where functions $f_{\beta_{i}}$ and $f_{\Delta }$, are provided in Equations (52) and (89).

The SROI is near-bounded (to the Z-axis of radial symmetry) by its vertices $P_{ns_{high}}$, $P_{ns_{mid}}$ and $P_{ns_{low}}$, which result from the following ray-intersection cases:

(a) $P_{ns_{high}}\leftarrow f_{\Delta }\left(\left(\theta_{1,max},\psi_{1}\right),\left(\theta_{2,max},\psi_{2}\right)\right)$
(b) $P_{ns_{mid}}\leftarrow f_{\Delta }\left(\left(\theta_{1,min},\psi_{1}\right),\left(\theta_{2,max},\psi_{2}\right)\right)$
(c) $P_{ns_{low}}\leftarrow f_{\Delta }\left(\left(\theta_{1,min},\psi_{1}\right),\left(\theta_{2,min},\psi_{2}\right)\right)$

where the intersection function $f_{\Delta }$ is implemented for direction rays (or angles) as defined in the Triangulation Section 5.2.

By assuming a radial symmetry on the camera’s field of view $\alpha_{cam}$, it should allow for a complete view of the mirror surface at its outmost diameter of $2r_{sys}$ according to Equation (6). Substantially, as depicted in Figure 6, $\alpha_{cam}$ is upper-bounded by the camera hole radius $r_{cam}$ selected according to Equation (78). The following inequality constraint emerges

(58) $$\begin{array}{c} 2\arctan\;\left(\frac{r_{sys}}{f_{z_{1}}\left(r_{sys}\right)}\right)\leq \alpha_{cam}\leq 2\arctan\;\left(\frac{r_{cam}}{f_{z_{2}}\left(r_{cam}\right)}\right) \end{array}$$

where the respective functions $f_{z_{i}}$ are defined in Equation (13).

**Figure 7:**
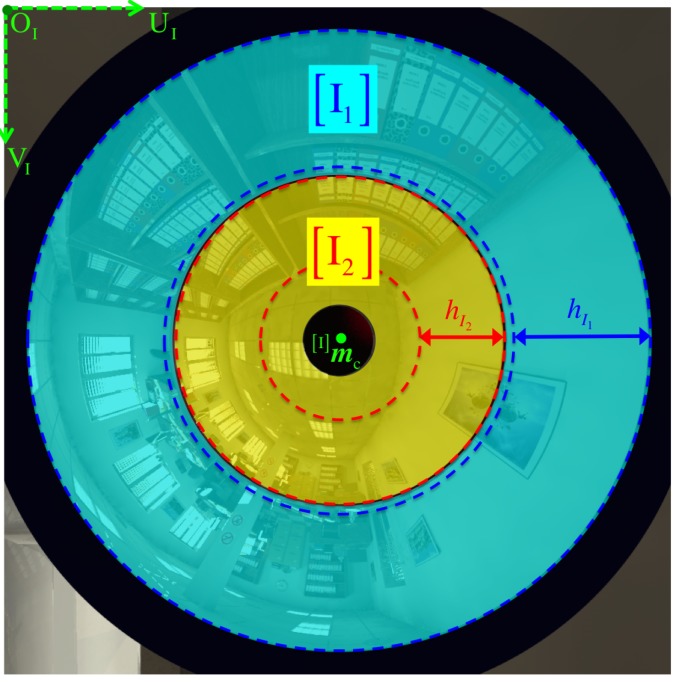
The omnidirectional image $\left[I\right]$ shown in Figure 2b is now annotated for the separate regions of interest in $\left[I_{1}\right]$ and $\left[I_{2}\right]$. In addition, we indicate the corresponding radial heights $h_{I_{1}}$ and $h_{I_{2}}$ of the SROI, so we can determine the imaging ratio $\chi_{I_{1:2}}=\frac{h_{I_{1}}}{h_{I_{2}}}$. For the optimal parameter values listed in Table 1, we find that $\chi_{I_{1:2}}\approx 2$.

Our specific viewing requirements when mounting the omnidirectional sensor along the central axis of the quadrotor ensure that objects located at 15 cm under the rig’s base and at 1 meter away (from the central axis) can be viewed. Thus, angles $\theta_{1,min}$ and $\theta_{2,min}$ should only be large enough as to avoid occlusions from the MAV’s propellers (Figure 5) and to produce inner and outer ring images at a useful ratio (Figure 7).

### 3.4. Spatial Resolution

The resolution of the images acquired by our system are not space invariant. In fact, an omnidirectional camera producing spatial resolution-invariant images can only be obtained through a non-analytical function of the mirror profile as shown in [31]. In this section, we study the effect our design has on its spatial resolution as it depends on position parameters like *d* and $c_{i}$ introduced in Section 2.1 as well as a direct dependency on the characteristics (e.g., focal length *f*) of the camera obtaining the image.

**Figure 8:**
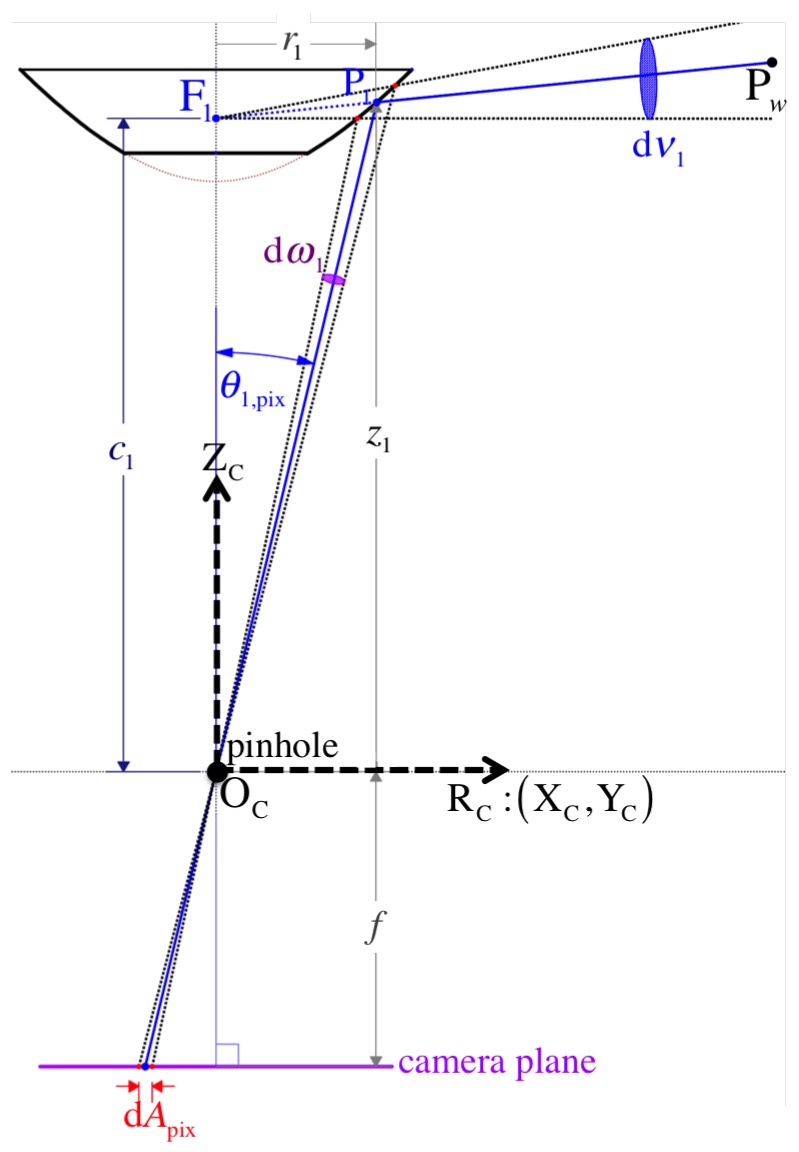
The spatial resolution for a central catadioptric sensor is the ratio between an infinitesimal image area d*A* and its corresponding solid angle d$\nu_{1}$ that views a point $P_{w}$. (Note: infinitesimal elements are exaggerated in the figure for better visualization.)

Let $\eta_{cam}$ be the spatial resolution for a conventional perspective camera as defined by Baker and Nayar in [25,29]. It measures the ratio between the infinitesimal solid angle d$\omega_{i}$ (usually measured in steradians) that is directed toward a point $P_{i}$ at an angle $\theta_{i,\mathrm{pix}}$ (formed with the optical axis $Z_{C}$) and the infinitesimal element of image area d$A_{\mathrm{pix}}$ that d$\omega_{i}$ subtends (as shown in Figure 8). Accordingly, we have:

(59) $$\begin{array}{cc} \eta_{cam} & =\frac{{dA}_{\mathrm{pix}}}{{d\omega }_{i}}=\frac{f^{2}}{\cos^{3}\theta_{i,\mathrm{pix}}} \end{array}$$

whose behavior tends to decrease as $\theta_{\mathrm{pix}}\to 0$, so higher resolution areas on the sensor plane continuously increase the farther away they get from the optical center imaged at ${}^{\left[I\right]}\;m_{c}$. For ease of visualization, we plot only the *u* pixel coordinates corresponding to the 2D spatial resolution $\eta_{2D}$, which is obtained by projecting the solid angle *Ω* onto a planar angle $\theta_{\Omega }$ (the apex angle in 2D of the solid cone of view). This yields $\theta_{\Omega }=2\arccos\;\left(1-\Omega /2\pi \right)$, and we reduce the image area into its circular diameter with $2\sqrt{A/\pi }$. Generally, our conversion from 3D spatial resolution *η* in $\left[m^{2}/\mathrm{sr}\right]$ units to 2D proceeds as follows:

(60) $$\begin{array}{c} \eta_{2D}=\frac{2\sqrt{\eta /\pi }}{\theta_{\Omega =1\mathrm{sr}}} \end{array}$$

where $\theta_{\Omega =1\mathrm{sr}}\approx 1.14390752211\mathrm{rad}$. More specifically, Equation (59) is manipulated to provide $\eta_{i,cam}$ as the indicative of spatial resolution toward any specific point in the mirror, ${}^{\left[C\right]}\;P_{i}\in M_{i}$ according to Equation (8), as follows:

(61) $$\begin{array}{cc} \eta_{i,cam} & =\left\{\begin{array}{cc} f^{2}{\left(\frac{\sqrt{r_{1}^{2}+z_{1}^{2}}}{z_{1}}\right)}^{3} & \;\text{if}\;i=1\\ f^{2}{\left(\frac{\sqrt{r_{2}^{2}+{\left(d-z_{2}\right)}^{2}}}{d-z_{2}}\right)}^{3} & \;\text{if}\;i=2 \end{array}\right. \end{array}$$

where $r_{i}$ is the radial length defined in Equation (6) and its associated $z_{i}$ coordinate, *f* is the camera’s focal length, and the design parameters *d* and $c_{i}$ that relate to the position of the mirror focal points $F_{i}$ with respect to the camera frame $\left[C\right]$.

Thus, for a conventional perspective camera, $\eta_{i,cam}$ grows as $\theta_{i,\mathrm{pix}}\to \pi /2$ due to the foreshortening effect that stretches the image representation around the sensor plane’s periphery where spatial information gets collected onto a larger number of pixels. Therefore, image areas farther from the optical axis are considered to have higher spatial resolutions.

Baker and Nayar also defined the resolution, $\eta_{i}$, of a catadioptric sensor in order to quantify the view of the world or d$\nu_{i}$, an infinitesimal element of the solid angle subtended by the mirror’s effective viewpoint $F_{i}$, which is consequently imaged onto a pixel area d$A_{\mathrm{pix}}$. Again, here we provide the resolution according to our model:

(62a) $$\begin{array}{cc} \eta_{1} & =\frac{{dA}_{\mathrm{pix}}}{{d\nu }_{1}}=\left[\frac{r_{1}^{2}+{\left(c_{1}-z_{1}\right)}^{2})}{r_{1}^{2}+z_{1}^{2}}\right]\eta_{1,cam} \end{array}$$

(62b) $$\begin{array}{cc} \eta_{2} & =\frac{{dA}_{\mathrm{pix}}}{{d\nu }_{2}}=\left[\frac{r_{2}^{2}+{\left(c_{2}-d+z_{2}\right)}^{2})}{r_{2}^{2}+{\left(d-z_{2}\right)}^{2}}\right]\eta_{2,cam} \end{array}$$

for our mirror-perspective camera configuration, where $O_{C}$ is the origin of coordinates as shown in Figure 8 and $\eta_{i,cam}$ is given in Equation (61).

As demonstrated by the plot of Figure 12 in Section 4.2.2, $\eta_{i}$ grows accordingly towards the periphery of each mirror (the equatorial region). This aspect of our sensor design is very important because it indicates that the common field of view, $\alpha_{SROI}$, where stereo vision is employed (Section 5), is imaged at a relatively higher resolution than the unused polar regions closer to the optical axis (the $Z_{C}$ axis).

If we modify $\eta_{i}$ by substituting $r_{i}$ with its equivalent $f_{r_{i}}\left(z_{i}\right)$ function defined in Equation (14), using mirror 1 for example, we get:

(63) $$\begin{array}{c} \begin{array}{cc} \eta_{1} & =\left[\frac{f_{r_{1}}^{2}\left(z_{1}\right)+{\left(c_{1}-z_{1}\right)}^{2}}{f_{r_{1}}^{2}\left(z_{1}\right)+z_{1}^{2}}\right]\eta_{1,cam}\\ & =\frac{f^{2}\sqrt{f_{r_{1}}^{2}\left(z_{1}\right)+z_{1}^{2}}\left[f_{r_{1}}^{2}\left(z_{1}\right)+{\left(c_{1}-z_{1}\right)}^{2}\right]}{z_{1}^{3}} \end{array} \end{array}$$

which is an inherent indicative of how the resolution $\eta_{i}$ for a reflection point $P_{i}$ increases with $k_{i}\to \infty$ (Figure 11). Conversely, the smaller the $k_{i}$ parameter gets (related to eccentricity as discussed in Section 2.2), the flatter the mirror becomes, so its resolution resembles more that of the perspective camera alone. Mathematically, $\lim_{k_{i}\to 2}\eta_{i}\to \eta_{i,cam}$.

**Figure 9:**
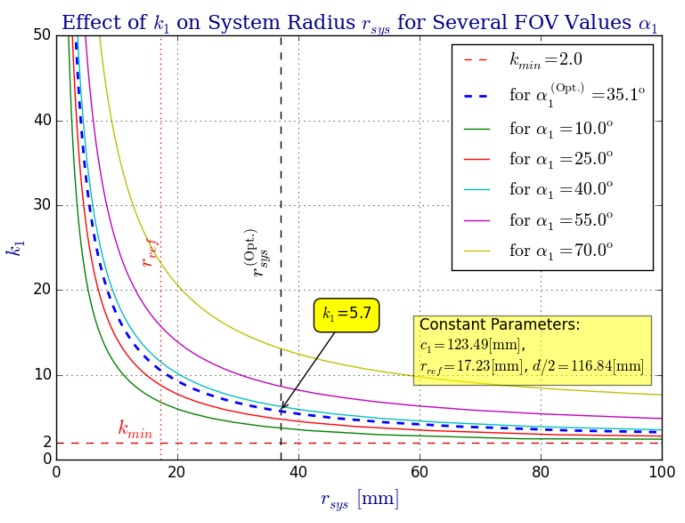
The effect that parameter $k_{i}$ (showing mirror 1 only) has over the system radius $r_{sys}$ for various values of the vertical field of view angle $\alpha_{1}$. In order to maintain a vertical field of view $\alpha_{i}$ that is bounded by $z_{max}{|}_{r_{sys}}$, the value of $r_{sys}$ must change accordingly. Inherently, the system’s height, $h_{sys}$, and its mass, $m_{sys}$, are also affected by $k_{i}$ (see Section 2.3).

**Figure 10:**
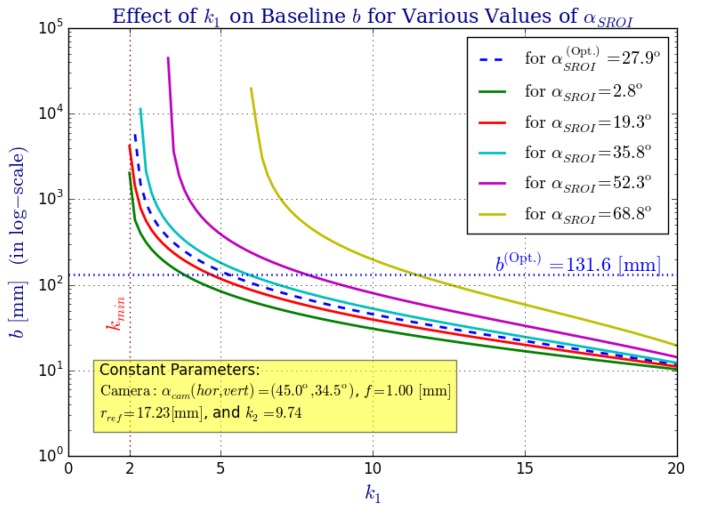
The effect that parameter $k_{1}$ has over the omnistereo system’s baseline *b* for several common FOV angles ($\alpha_{SROI}$) and a fixed camera with $\alpha_{cam}$. An inverse relationship exists between *k* and *b* as plotted here (using a logarithmic scale for the vertical axis). Intuitively, the flatter the mirror gets (k→2), the farther $F_{1}$ must be translated in order to fit within the camera’s view, $\alpha_{SROI}$, causing *b* to increase.

As shown in Figure 9, a smaller $k_{i}$ would require a wider radius $r_{sys}$ in order to achieve the same omnidirectional vertical field of view, $\alpha_{sys}$. Even worse, in order to image such a wider reflector, either the camera’s field of view, $\alpha_{cam}$, would have to increase (by decreasing the focal length *f* and perhaps requiring a larger camera hole $r_{cam}$ and sensor size), or the distance $c_{i}$ between the effective pinhole and the viewpoint would have to increase accordingly. Another consequence is the effect on the baseline *b*, which must change in order to maintain the same vertical field of view (Figure 10). As a result, the depth resolution of the stereo system would suffer as well.

## 4. Parameter Optimization and Prototyping

The nonlinear nature of this system makes it very difficult to balance among its desirable performance aspects. The optimal vector of design parameters, $\theta^{*}$, can be found by posing a constrained maximization problem for the objective function

(64) $$\begin{array}{c} f_{b}\left(\theta \right)=c_{1}+c_{2}-d \end{array}$$

which measures the baseline according to Equation (3). Indeed, the optimization problem is subject to the set of constraints *C*, which we enumerate in Section 4.1. Formally,

(65) $$\begin{array}{c} \theta^{*}=\underset{\theta \in \Theta }{\mathrm{arg max}}f_{b}\left(\theta \right)\;\;\text{subject}\;\text{to}\;C \end{array}$$

where $\Theta \subseteq R^{6}$ is the 6-dimensional solution space for $\theta \in R^{6}$ given in Equation (4) as $\theta =\left[\begin{array}{cccccc} c_{1}, & c_{2}, & k_{1}, & k_{2}, & d, & r_{sys} \end{array}\right]$.

### 4.1. Optimization Constraints

We discuss the constraints that the proposed omnistereo sensor is subject to. Overall, we mainly take the following into account:

(a) *geometrical constraints* — including SVP and reflex constraints described by Equations (11), (12) and (2);
(b) *physical constraints* — the rig’s dimensions, which include the mirrors radii as well as by-product parameters such as system height $h_{sys}$ and mass $m_{sys}$;
(c) *performance constraints* — the spatial resolution and range from triangulation determined by parameters $k_{1}$, $k_{2}$, and $c_{1}$; the desired viewing angles for an optimal SROI field of view, $\alpha_{SROI}$.

Following the design model described throughout Section 2, we now list the pertaining linear and nonlinear constraints that compose the set *C*. We disjoint the linear constraints in a subset $C_{L}$ and the non-linear constraints subset $C_{NL}$, so $C=C_{L}⊎C_{NL}$. Within each subset, we generalize equality constraints as functions $h:R^{6}\mapsto R$ that obey

(66) $$\begin{array}{c} h(\theta )=0 \end{array}$$

whereas inequality functions $g:R^{6}\mapsto R$ satisfy

(67) $$\begin{array}{c} g(\theta )\leq 0 \end{array}$$

#### 4.1.1. Linear Constraints

We have only setup linear inequalities for constraints in $C_{L}$. Specifically, we require the following:

g1: In order to set the position of $F_{2}$ below the origin $O_{C}$ of the pinhole camera frame $\left[C\right]$, the focal distance $c_{2}$ of mirror 2 must be larger than *d* (distance between $O_{C}$ and $F_{2v}$),
  (68) $$\begin{array}{c} d\leq c_{2} \end{array}$$
g2: Because the hyperboloidal mirror should reflect light towards its effective viewpoint $F_{1}$ without being occluded by the reflex mirror, mirror 1’s focal distance, $c_{1}$, needs to exceed the placement of the reflex mirror,
  (69) $$\begin{array}{c} d/2\leq c_{1} \end{array}$$
g3: The empirical constraint
  (70) $$\begin{array}{c} \frac{5}{3}\leq \frac{k_{2}}{k_{1}} \end{array}$$
  pertains our rig dimensions in order to assign a greater curvature to mirror 2’s profile (located a the bottom), so its view is directed toward the equatorial region rather than up. Complementarily, this constraint flattens mirror 1’s profile, so it can possess a greater view of the ground. This curvature inequality allows the SROI to be bounded by a wider vertical field of view when the sensor must be mounted above the MAV’s propellers as depicted in Figure 5.

#### 4.1.2. Non-Linear Constraints

For the non-linear design constraints, we establish the following inequalities:

g4: The AscTec Pelican quadrotor has a maximum payload of 650g (according to the manufacturer specifications [28]). Therefore, we must satisfy the system mass computed via Equation (16), such that
  (71) $$\begin{array}{c} m_{sys}\leq 650 \end{array}$$
g5: Similarly, we limit the system’s height obtained with Equation (15) by a height limit $h_{sys,max}$,
  (72) $$\begin{array}{c} h_{sys}\leq h_{sys,max} \end{array}$$
  For example, we set $h_{sys,max}=150\mathrm{mm}$ for the 37mm-radius rig.
g6: The origin of coordinates for the camera frame is set at its viewpoint, $O_{C}$. In order to fit the camera enclosure under mirror 2, it is realistic to position the focus $F_{2}$ on the vertical transverse axis at more than 5mm away from $O_{C}$:
  (73) $$\begin{array}{c} 5\leq z_{0_{2}}-a_{2} \end{array}$$
  where $z_{0_{2}}$ is defined in Equation (10), and $a_{2}$ pertains to Equation (5).

Next, we determine the bounds for the limiting angles that partake in the computation of the system’s vertical field of view $\alpha_{sys}$, which is based on equation Equation (54). Our application has specific viewing requirements that can be achieved with the following application conditions:

g7: Let $\Lambda_{1,max}=14^{\circ }$ be an acceptable upper-bound for angle $\theta_{1,max}$ , such that
  (74) $$\begin{array}{c} \theta_{1,max}\leq \Lambda_{1,max} \end{array}$$
g8: Because we desire a larger view towards the ground from mirror 1, we empirically set $\Lambda_{1,min}=-25^{\circ }$ as a lower-bound for the minimum elevation $\theta_{1,min}$,
  (75) $$\begin{array}{c} \Lambda_{1,min}\leq \theta_{1,min} \end{array}$$
g9: In order to avoid occlusions with the MAV’s propellers while being capable to image objects located about 5 cm under the rig’s base and 20 cm away (horizontally) from the central axis, we limit mirror 2’s lowest angle by a lower-bound $\Lambda_{2,min}=-14^{\circ }$,
  (76) $$\begin{array}{c} \Lambda_{2,min}\leq \theta_{2,min} \end{array}$$

Finally, we restrict the radius of the system, $r_{sys}$, to be identical for both hypeboloids by satisfying the following equality condition:

h1: With functions $f_{r_{1}}$ and $f_{r_{2}}$ defined in Equation (14), we set
  $$\begin{array}{cc} r_{sys}=r_{i,max} & =f_{r_{i}}\left(z_{i,max}\right),\forall i\in \left\{1,2\right\} \end{array}$$
  where we imply that $z_{i,max}\leftarrow f_{z_{i}}\left(r_{sys}\right)$ using Equation (13). Thus, the entire function composition for this equality becomes
  (77) $$\begin{array}{cc} f_{r_{1}}\left(f_{z_{1}}\left(r_{sys}\right)\right) & =f_{r_{2}}\left(f_{z_{2}}\left(r_{sys}\right)\right) \end{array}$$

### 4.2. Optimal Results

Applying the aforementioned constraints (Section 4.1) and using an iterative nonlinear optimization method such as one of the surveyed in [32], a bounded solution vector $\theta^{*}$ converges to the the values shown in Table 1 for two rig sizes. Table 2 contains the by-product parameters corresponding to the dimensions listed in Table 1.

As Figure 3 illustrates, a realistic dimension for the radius of the camera hole, $r_{cam}$, must consider the maximum value between a physical micro-lens radius ($r_{lens}$) and the radius $r_{\alpha_{cam}{|}_{r_{sys}}}$ for an unoccluded field of view of the camera $\alpha_{cam}$ imaging the complete surface of mirror 1. Practically,

(78) $$r_{cam}=\max\left(r_{lens},r_{\alpha_{cam}{|}_{r_{sys}}}\right)$$

For both rigs, the expected vertical field of views are $\alpha_{sys}=75{}^{\circ}-\left(-21{}^{\circ}\right)\approx 96{}^{\circ}$ according to Equation (54), and $\alpha_{SROI}=14{}^{\circ}-\left(-14{}^{\circ}\right)\approx 28{}^{\circ}$ using Equation (55). Note that $\theta_{2,max}$ may be actually limited by the camera hole radius, so in reality $\theta_{cam}⇝59{}^{\circ}$, and $\alpha_{sys}⇝80{}^{\circ}$. For the big rig, Table 3 shows the nearest vertices of the SROI that result from these angles (Figure 6).

#### 4.2.1. Optimality of Parameters $k_{1}$ and $k_{2}$

**Figure 11:**
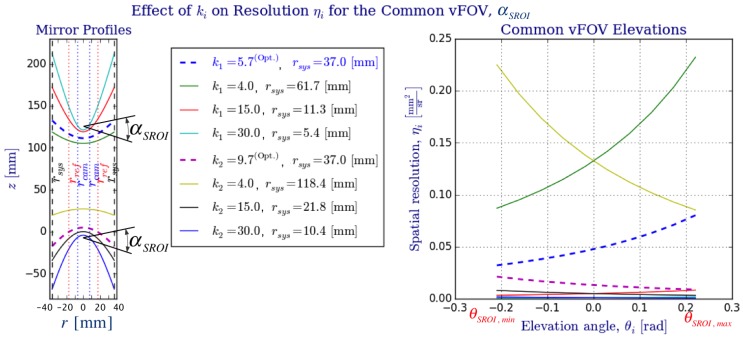
Comparison of $k_{i}$ values and their effect on spatial resolution $\eta_{i}$ for i={1,2}. For the big rig, the optimal focal dimensions $c_{1}$ and $c_{2}$ (from Table 1) were used as well as the angular span on the common vertical FOV, $\alpha_{SROI}\approx 28$. Although resolution $\eta_{i}^{\left(Opt.\right)}$ for the optimal values of $k_{i}$ could be improved by employing smaller *k* values (lower curvature profiles indicated on the left plot of the figure), this would in turn increase the system radius, $r_{sys}$, as to maintain $\alpha_{i}$ (Figure 9). As expected, the plot on the right help us appreciate how the spatial resolutions, $\eta_{i}$, increase towards the equatorial regions ($\theta_{1}\to \theta_{SROI,max}$ and $\theta_{2}\to \theta_{SROI,min}$).

**Figure 12:**
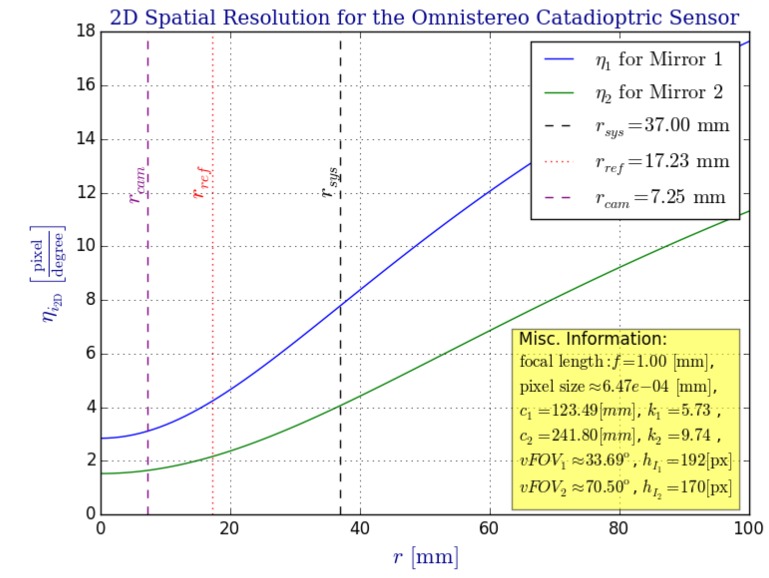
Using the formula given in Equation (60), we plot the 2D version of the spatial resolution of our proposed omnistereo catadioptric sensor (37mm-radius rig). Both resolutions $\eta_{1}$ and $\eta_{2}$ increase towards the equatorial region where they are physically limited by $r_{sys}$. This verifies the spatial resolution theory given in [29], and it justifies our coaxial configuration useful for omnistereo sensing within the SROI indicated in Figure 6.

Finally, we study the effect parameter $k_{i}$ has over the system radius $r_{sys}$ (Figure 9), the omnistereo baseline *b* (Figure 10), and the spatial resolution (Figure 11 and Figure 12). Figure 9 addresses the relation between $k_{i}$ and radius $r_{sys}$ (recall the rig size specified in Section 2.3). In Figure 11, it can be seen that for the same $r_{sys}$, realistic values for $k_{1}$ fall in the range $3<k_{1}<13$, and the vertical field of view $\alpha_{1}\to 0$ as k→2, which is expected according to the SVP property specified in Section 2.2. In fact, the left part of Figure 11 also demonstrates the necessary $r_{sys}$ to maintain $\alpha_{SROI}\approx 28$ for various values of $k_{i}$.

Figure 10 shows the inverse relationship between values of $k_{1}$ and the baseline, *b*, as we attempt to fit the view of a wider/narrower mirror profile (due to $k_{1}$) on the constant camera field of view, $\alpha_{cam}$. In order to make a fair comparison, let

$$k_{1}^{\prime }=k_{1}+\varepsilon_{k},\;\forall k_{1}>2,\varepsilon_{k}>0$$

for which we find its new focal length $c_{1}^{\prime }$ while solving for the new $r_{sys}^{\prime }$ and $z_{max}^{\prime }$. Provided with a function such that $c_{1}\leftarrow f_{c_{1}}\left(k_{1}\right)$, we perform the analysis for a given $\alpha_{SROI}$ and $\alpha_{cam}$ shown in Figure 10. Given the baseline function $f_{b}$ defined in Equation (64), the following implication holds true:

(79) $$\begin{array}{c} f_{b}{|}_{c_{1}\leftarrow f_{c_{1}}\left(k_{1}\right)}>f_{b}{|}_{c_{1}^{\prime }\leftarrow f_{c_{1}}\left(k_{1}+\varepsilon_{k}\right)},\;\forall k_{1}>2,\varepsilon_{k}>0 \end{array}$$

Notice that $k_{2}$, $c_{2}$ and *d* are kept constant through this last analysis, and we ignore possible occlusions from the reflex mirror fixed at d/2.

#### 4.2.2. Spatial Resolution Optimality

In this section, we compare the sensor’s spatial resolution, $\eta_{i}$, defined in Section 3.4 for the optimal parameters listed in Table 1 (for the big rig, only). In Figure 12, we verify how both resolutions $\eta_{1}$ and $\eta_{2}$ increase towards the equatorial region according to the spatial resolution theory presented in [29]. Indeed, the increase in spatial resolution within the SROI that covers the equatorial region (as indicated in Figure 6) justifies our model’s coaxial configuration intended for omnistereo applications.

In Figure 11, we compare the effect on $\eta_{i}$ for various mirror profiles, which depend directly on $k_{i}$. We illustrate the change in curvature due to parameters $k_{1}$ and $k_{2}$ and also show (in the legend) the respective $r_{sys}$ achieving a common vFOV of $\alpha_{SROI}\approx 28{}^{\circ}$ as for the optimal parameters of the big rig. From this plot, we appreciate the compromise due to optimal parameters, $k_{1}^{\left(Opt.\right)}=5.7$ and $k_{2}^{\left(Opt.\right)}=9.7$, for a realistic system size due to $r_{sys}$ and a suitable range of spatial resolutions, $\eta_{i}$, within the SROI.

### 4.3. Prototypes

We validate our design with both synthetic and real-life models.

#### 4.3.1. Synthetic Prototype (Simulation)

After converging to an optimal solution $\theta^{*}$, we employ these parameters (Table 1) to describe synthetic models using POV-Ray, an open-source ray-tracer. We render 3D scenes via the camera of the synthetic omnistereo sensor like the example shown in Figure 2b. The simulation stage plays two important roles in our investigation:

(1) to acquire ground-truth 3D-scene information in order to evaluate the computed range by the omnistereo system (as explained in Section 5); and
(2) to provide an almost accurate geometrical representation of the model by discounting some real-life computer vision artifacts such as assembly misalignments, glare from the support tube (motivating the use of standoffs on the real prototype), as well as the camera’s shallow depth-of-field. All of these artifacts can affect the quality of the real-life results shown in Section 6.

#### 4.3.2. Real-Life Prototypes

**Figure 13:**
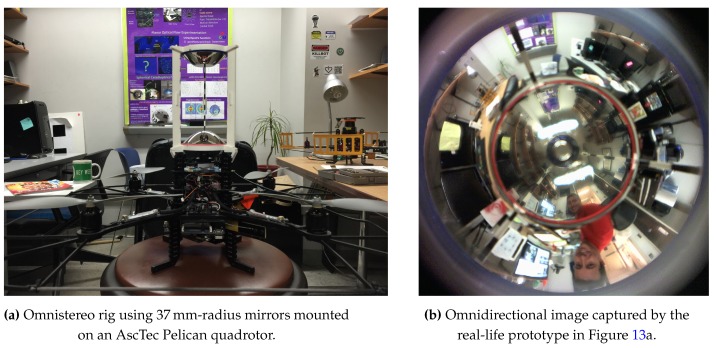
Real-life prototype of the omnistereo sensor.

We have also produced two physical prototypes that can be installed on the Pelican quadrotor (made by Ascending Technologies [28]). Figure 13a shows the rig constructed with hyperboloidal mirrors of $r_{sys}\approx 37\mathrm{mm}$, and a Logitech^®^ HD Pro Webcam C910 camera capable of (2592 × 1944) pixel images at 15∼20 FPS. We decided to skip the use of the acrylic glass tube to separate the mirrors at the specified $h_{sys}$ distance, and instead we constructed a lighter 3-standoff mount in order to avoid glare and cross-reflections. This support was designed in 3D-CAD and printed for assembly. The three areas of occlusion due to the 3mm-wide standoffs are non-invasive for the purpose of omnidirectional sensing and can be ignored with simple masks during image processing. In fact, we stamped fiducial markers to the vertical standoffs to aid with the panoramas generation (Section 5.1) and future calibration methods. To image the entire surface of mirror 1, we require a camera with a (minimum) field of view of $\alpha_{cam}>31{}^{\circ}$, which is achieved by $r_{\alpha_{cam}}>1.4\mathrm{mm}$. In practice, as noted by Equation (78), microlenses measure around $r_{lens}\approx 7\mathrm{mm}$. Therefore, we set $r_{cam}>7\mathrm{mm}$, as a safe specification to fit a standard microlens through the opening of mirror 2 as shown in Figure 3.

Recall that $m_{sys}$ is limited by the maximum 650g-payload that the AscTec Pelican quadrotor is capable of flying with (according to the manufacturer specifications [28]). The camera with lens weights approximately 25g. A cylindrical tube made of acrylic has an average density $\rho_{tub}\approx 1.18$ g·cm${}^{-3}$, whereas the mirrors machined out of brass have a density $\rho_{mir}\approx 8.5$ g·cm${}^{-3}$. Empirically, we verify a close estimate of the entire system’s mass, such that $m_{sys}\approx 550g$ for the big rig, and $m_{sys}\approx 150g$ for the small rig.

## 5. 3D Sensing from Omnistereo Images

Stereo vision from point correspondences on images at distinct locations is a popular method for obtaining 3D range information via triangulation. Techniques for image point matching are generally divided between dense (area-based scanning [32]) and sparse (feature description [33]) approaches. Due to parallax, the disparity in point positions for objects close to the vision system must be larger than for objects that are farther away. As illustrated in Figure 6, the nearsightedness of the sensor is determined mainly by the common observable space (a.k.a. SROI) acquired by the limiting elevation angles of the mirrors (Section 3.3). In addition, we will see next (Section 5.2) that the baseline *b* also plays a major role in range computation.

Due to our model’s coaxial configuration, we could scan for pixel correspondences radially between a given pair of warped images $\left(\left[I_{1}\right],\left[I_{2}\right]\right)$ like in the approach taken by similar works such as [34]. However, it seems more convenient to work on a rectified image space, such as with panoramic images, where the search for correspondences can be performed using any of the various existing methods for perspective stereo views. Hence, we first demonstrate how these rectified panoramic images are produced (Section 5.1) and used for establishing point correspondences. Then, we proceed to study our triangulation method for the range computation from a given set of point correspondences (Section 5.2). Last, we show preliminary 3D point clouds as the outcome from such procedure.

### 5.1. Panoramic Images

**Figure 14:**
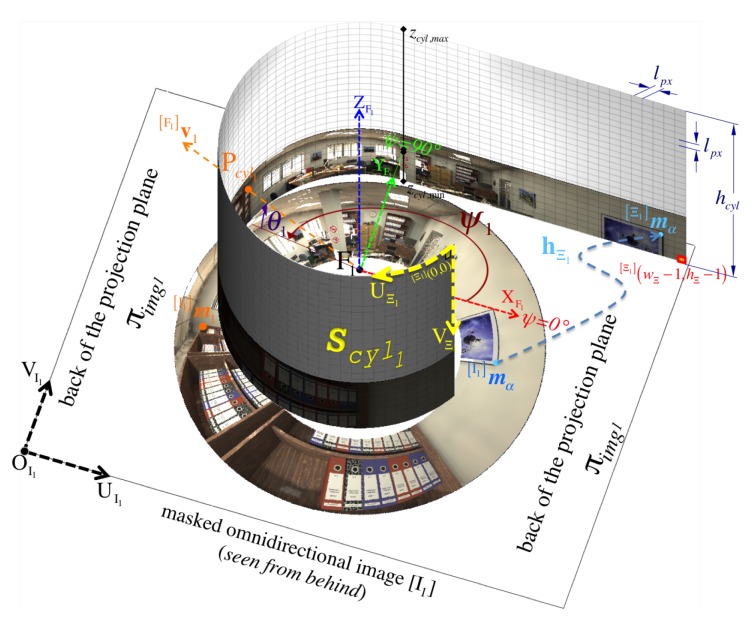
An example for the formation of panoramic image $\left[\Xi_{1}\right]$ out of the omnidirectional image $\left[I_{1}\right]$ (showing only the masked region of interest on the back of image plane $\pi_{img_{1}}$). Any particular ray, $v_{1}$ indicated by its elevation and azimuth such as ${}^{\left[F_{1}\right]}\;\left(\psi_{1},\theta_{1}\right)$ that is directed towards the focus $F_{1}$ must traverse the projection cylinder $S_{cyl_{1}}$ at point $P_{cyl_{1}}$. More abstractly, the figure also shows how a pixel position ${}^{\left[\Xi_{1}\right]}\;m_{\alpha }$ on the panoramic pixel space gets mapped from its corresponding pixel position ${}^{\left[I_{1}\right]}\;m_{\alpha }$ via function $h_{\Xi_{1}}$ defined in Equation (85). Although not up to scale, it’s crucial to notice the relative orientation between $S_{cyl_{1}}$ and the *back* of the projection plane $\pi_{img_{1}}$ where the omnidirectional image $\left[I_{1}\right]$ is found.

Figure 14 illustrates how we form the respective panoramic image $\left[\Xi_{1}\right]$ out of its warped omnidirectional image $\left[I_{1}\right]$. As illustrated in Figure 7, $\left[I_{i}\right]$ is simply the region of interest out of the full image $\left[I\right]$ where projection occurs via mirror *i*. However, we can safely refer to $\left[I\right]$ because it will never be the case that projections via different mirrors overlap on the same pixel position ${}^{\left[I\right]}\;m$. In a few words, we obtain a panorama $\left[\Xi_{i}\right]$ by reverse-mapping each discretized 3D point $P_{cyl_{i}}\in S_{cyl_{i}}$ to its projected pixel coordinates ${}^{\left[I\right]}\;m$ on $\left[I\right]$ according to Section 3.2.

More thoroughly, for i={1,2}, $S_{cyl_{i}}$ is the set of all valid 3D points $P_{cyl_{i}}$ that lie on an imaginary unit cylinder centered along the Z-axis and positioned with respect to the mirror’s primary focus $F_{i}$. Recall that the radius of a unit cylinder is $r_{cyl}=1$, so its circumference becomes $w_{cyl}=2\pi r_{cyl}=2\pi$. Noticed that the imaging ratio, $\chi_{I_{1:2}}=\frac{h_{I_{1}}}{h_{I_{2}}}$, illustrated in Figure 7 provides a way of inferring the scale between pairs of point correspondences. However, we achieve conforming scales among both panoramic representations by simply setting both cylinders to an equal height $h_{cyl}$, which is determined from the system’s elevation limits, $\left(\theta_{sys,min},\theta_{sys,max}\right)$, since they partake in the measurement of the system’s vertical field of view given by Equation (54). Hence, we obtain

(80) $$\begin{array}{c} h_{cyl}=z_{cyl,max}-z_{cyl,min}\;,\;\text{where}\;\left\{\;\begin{array}{cc} z_{cyl,max} & =\tan\left(\theta_{sys,max}\right)\\ z_{cyl,min} & =\tan\left(\theta_{sys,min}\right) \end{array}\right. \end{array}$$

Consequently, to achieve panoramic images $\left[\Xi_{i}\right]$ of the same dimensions by maintaining a true aspect ratio $w_{\Xi }:h_{\Xi }$, it suffices to indicate either the width (number of columns) $w_{\Xi }$ or the height (number of rows) $h_{\Xi }$ as number of pixels. Here, we propose a custom method for resolving the panoramic image dimensions by setting the equality for the length $l_{px}$ of an individual “square” pixel in the cylinder (behaving like a panoramic camera sensor):

(81) $$\begin{array}{c} l_{px}=\frac{w_{cyl}}{w_{\Xi }}=\frac{h_{cyl}}{h_{\Xi }} \end{array}$$

For instance, if the width $w_{\Xi }$ is given, then the height is simply $h_{\Xi }=w_{\Xi }h_{cyl}/w_{cyl}$.

To increase the processing speed for each panoramic image $\left[\Xi_{i}\right]$, we fill up its corresponding look-up-table LUT${}_{\Xi_{i}}$ of size $w_{\Xi }\times h_{\Xi }$ that encodes the mapping for each panoramic pixel coordinates ${}^{\left[\Xi_{i}\right]}\;m={}^{\left[\Xi_{i}\right]}\;{\left[\begin{array}{cc} u, & v \end{array}\right]}^{T}$ to its respective projection ${}^{\left[I_{i}\right]}\;m={}^{\left[I_{i}\right]}\;{\left[\begin{array}{cc} u, & v \end{array}\right]}^{T}$ on the distorted image $\left[I_{i}\right]$. Each pixel ${}^{\left[\Xi_{i}\right]}\;m$ gets associated with its cylinder’s 3D point positioned at ${}^{\left[F_{i}\right]}\;p_{cyl_{i}}$, which can inherently be indicated by its elevation ${}^{\left[F_{i}\right]}\;\theta_{i}$ and azimuth ${}^{\left[F_{i}\right]}\;\psi_{i}$ (relative to the mirror’s primary focus $F_{i}$) as illustrated in Figure 4. Thus, the ray ${}^{\left[F_{i}\right]}\;v_{i}$ of a particular 3D point directed about ${}^{\left[F_{i}\right]}\;\left(\psi_{i},\theta_{i}\right)$ must pass through $P_{cyl_{i}}$ in order to get imaged as pixel ${}^{\left[I\right]}\;m_{i}$.

Since the circumference of the cylinder, $w_{cyl}$, is discretized with respect to the number of pixel columns or width $w_{\Xi }$, we use the pixel length $l_{px}$ as the factor to obtain the arc length $l_{\psi_{i}}$ spanned by the azimuth ${}^{\left[F_{i}\right]}\;\psi_{i}$ out of a given ${}^{\left[\Xi_{i}\right]}\;u$ coordinate on the panoramic image. Generally,

(82) $$\begin{array}{c} {}^{\left[F_{i}\right]}\;\psi_{i}=\frac{l_{\psi_{i}}}{r_{cyl}}=\frac{w_{cyl}-{}^{\left[\Xi_{i}\right]}\;u\;l_{px}}{r_{cyl}} \end{array}$$

or simply ${}^{\left[F_{i}\right]}\;\psi_{i}=2\pi -{}^{\left[\Xi_{i}\right]}\;u\;l_{px}$ for the unit cylinder case.

An order reversal in the columns of the panorama is performed by Equation (82) because we account for the relative position between $S_{cyl_{i}}$ and the projection plane $\pi_{img}$. For $\left[\Xi_{1}\right]$, Figure 14 depicts the unrolling of the cylindrical panoramic image onto a planar panoramic image. However, note that $\pi_{img}$ is shown from above (or its back) in Figure 14, so the panorama visualization places the viewer inside the cylinder at $F_{1}$.

Similarly, the elevation angle ${}^{\left[F_{i}\right]}\;\theta_{i}$ is inferred out the row or ${}^{\left[\Xi_{i}\right]}\;v$ coordinate, which is scaled to its cylindrical representation by $l_{px}$. Recall that both cylinders have the same height, $h_{cyl}$, computed by Equation (80). By taking into account any row offset from the maximum height position, ${}^{\left[F_{i}\right]}\;z_{cyl,max}$, of the cylinder, we get

(83) $$\begin{array}{cc} {}^{\left[F_{i}\right]}\;\theta_{i} & =\arctan\;\left({}^{\left[F_{i}\right]}\;z_{cyl,max}-{}^{\left[\Xi_{i}\right]}\;v\;l_{px}\right) \end{array}$$

Given these angles and assuming coaxial alignment, we evaluate the positon vector ${}^{\left[C\right]}\;p_{cyl_{i}}$ for a point on the panoramic cylinder with respect to the camera frame $\left[C\right]$:

(84) Cpcyli=rcylcosFiψisinFiψitanFiθi+Cfi

where $r_{cyl}$ cancels out for a unit cylinder. The direction equations Equations (82) and (83) leading to Equation (84) as a process: ${}^{\left[\Xi_{i}\right]}\;m\overset{\;\text{Equation}\;(82)\;}{\underset{\;\text{Equation}\;(83)\;}{\mapsto }}{}^{\left[F_{i}\right]}\;\left(\begin{array}{c} \psi \\ \theta \end{array}\right)\overset{\;\text{Equation}\;(84)\;}{\mapsto }{}^{\left[C\right]}\;p_{cyl_{i}}$, which is eventually used as the input argument to Equation (36) in order to determine pixel ${}^{\left[I_{i}\right]}\;m$ via the mapping function $h_{\Xi_{i}}:R^{2}\mapsto R^{2}$,

(85) $$\begin{array}{ccc} {}^{\left[I_{i}\right]}\;m\leftarrow h_{\Xi_{i}}\left({}^{\left[\Xi_{i}\right]}\;m\right) & := & f_{\varphi_{i}}\left({\left.{}^{\left[C\right]}\;p_{cyl_{i}}\right|}_{\;{}^{\left[\Xi_{i}\right]}\;m}\right) \end{array}$$

#### Stereo Matching on Panoramas

**Figure 15:**
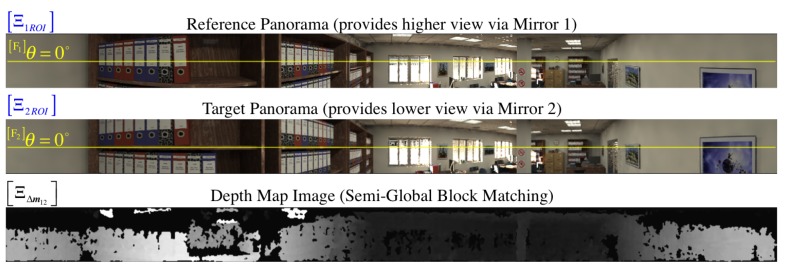
For the synthetic omnidirectional image $\left[I\right]$ shown in Figure 2b, we generate its pair of panoramic images $\left(\left[\Xi_{1}\right],\left[\Xi_{2}\right]\right)$ using the procedure explained in Section 5.1. Note that we only work on the SROI (shown here) to perform a semi-global block match between the panoramas as indicated in Section 5.1.1. The resulting disparity map, $\left[\Xi_{\Delta m_{12}}\right]$, is visualized at the bottom as a gray-scale panoramic image normalized about its 256 intensity levels, where brighter colors imply larger disparity values. To distinguish the relative vertical view of both panoramas, we have annotated the row position of the zero-elevation.

We understand that the algorithm chosen for finding matches is crucial to attain correct pixel disparity results. We refer the reader to [35] for a detailed survey of stereo correspondence methods. After comparing various block matching algorithms, we were able to obtain acceptable disparity maps with the semi-global block matching (SGBM) method introduced by [36], which can find subpixel matches in real time. As a result of this stereo block matcher among the pair of panoramic images $\left(\left[\Xi_{1}\right],\left[\Xi_{2}\right]\right)$, we get the dense disparity map $\left[\Xi_{\Delta m_{12}}\right]$ visualized as an image in Figure 15 and Figure 21a. Note that valid disparity values must be positive $\left({\left.\Delta m_{12}\right|}_{\;{}^{\left[\Xi_{i}\right]}\;m_{1}}>0\right)$ and they are given with respect to the reference image, in this case, $\left[\Xi_{1}\right]$. In addition, recall that no stereo matching algorithm (as far as we are aware) is totally immune to mismatches due to several well-known reasons in the literature such as ambiguity of cyclic patterns.

An advantage of the block (window) search for correspondences is that it can be narrowed along epipolar lines. Unlike the traditional horizontal stereo configuration, our system captures panoramic images whose views differ in a vertical fashion. As shown in [14], the unwrapped panoramas contain vertical, parallel epipolar lines that facilitate the pixel correlation search. Thus, given a pixel position ${}^{\left[\Xi_{i}\right]}\;m_{1}$ on the reference panorama $\left[\Xi_{1}\right]$ and its disparity value ${\left.\Delta m_{12}\right|}_{\;{}^{\left[\Xi_{i}\right]}\;m_{1}}$, we can resolve the correspondence ${}^{\left[\Xi_{2}\right]}\;m_{2}$ pixel coordinate on the target image, $\left[\Xi_{2}\right]$, by simply offsetting the *v*-coordinate with the disparity value:

(86) $$\begin{array}{cc} {}^{\left[\Xi_{2}\right]}\;m_{2} & =\left[\begin{array}{c} u_{1}\\ v_{1}+{\left.\Delta m_{12}\right|}_{\;{}^{\left[\Xi_{1}\right]}\;m_{1}} \end{array}\right] \end{array}$$

### 5.2. Range from Triangulation

Recall the duality that states a point $P_{w}$ as the intersection of a pair of lines. Regardless of the correspondence search technique employed, such as block stereo matching between panoramas $\left[\Xi_{i}\right]$ (Section 5.1.1) or feature detection directly on $\left[I\right]$, we can resolve for ${}^{\left[I\right]}\;\left(m_{1},m_{2}\right)$. From Equations (42) and (49), we obtain the respective pair of back-projected rays $\left({}^{\left[F_{1}\right]}\;v_{1},{}^{\left[F_{2}\right]}\;v_{2}\right)$, emanating from their respective physical viewpoints, $F_{1}$ and $F_{2}$, which are separated by baseline *b*. We can compute elevation angles $\theta_{1}$ and $\theta_{2}$ using equations Equations (43) and (50). Then, we can triangulate the back-projected rays in order to calculate the horizontal range $\rho_{w}$ defined in Equation (22), as follows:

(87) $$\rho_{w}=\frac{b\cos\left(\theta_{1}\right)\cos\left(\theta_{2}\right)}{\sin(\theta_{1}-\theta_{2})}$$

Finally, we obtain the 3D position of $P_{w}$:

(88) $${}^{\left[C\right]}\;p_{w}=\left[\begin{array}{c} -\rho_{w}\cos\left(\psi_{12}\right)\\ -\rho_{w}\sin\left(\psi_{12}\right)\\ c_{1}-\rho_{w}\tan\left(\theta_{1}\right) \end{array}\right]$$

where $\psi_{12}$ is the common azimuthal angle (on the XY-plane) for coplanar rays, so it can be determined either by Equation (44) or Equation (51). Functionally, we define the “naive” intersection function that implements Equations (87) and (88) such that

(89) $$\begin{array}{c} {}^{\left[C\right]}\;p_{w}\leftarrow f_{\Delta }\left(\left(\theta_{1},\psi_{1}\right),\left(\theta_{2},\psi_{2}\right),\theta \right) \end{array}$$

where **θ** is the model parameters vector defined in Equation (4) and can be omitted when calling this function because the model parameters should not change (ideally).

#### 5.2.1. Common Perpendicular Midpoint Triangulation Method

**Figure 16:**
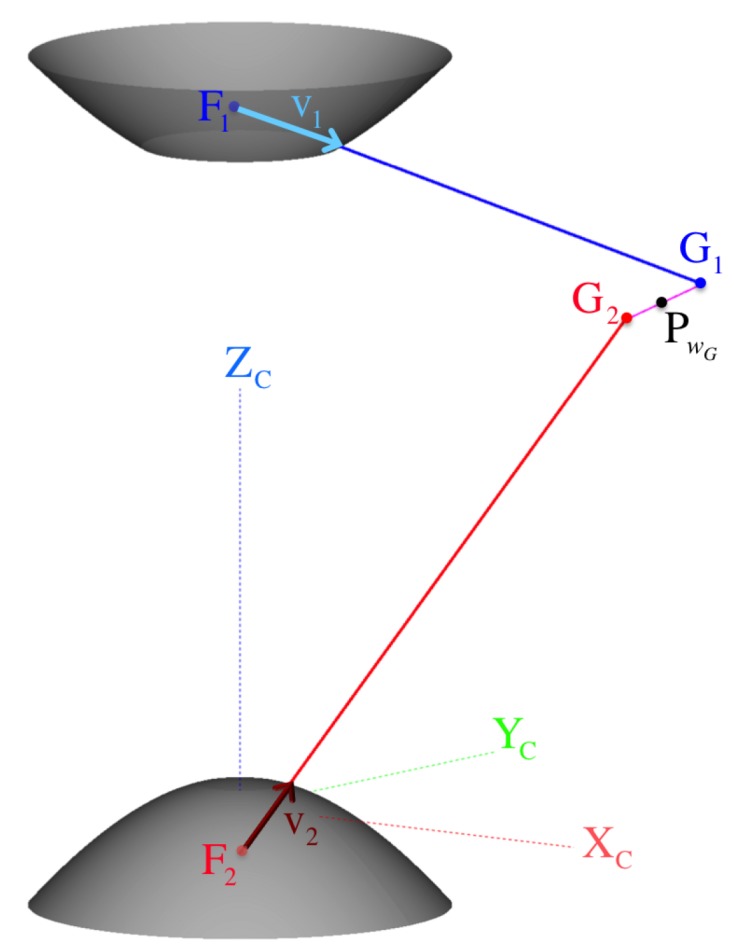
The more realistic case of skew back-projection rays $\left(v_{1},v_{2}\right)$ approximates the triangulated point $P_{w}$ by getting the midpoint $P_{w_{G}}$ on the common perpendicular line segment $\bar{G_{1}G_{2}}:\lambda_{1⊥2}{\hat{v}}_{1⊥2}$. Note that the visualized skew rays were formed from a pixel correspondence pair ${}^{\left[I\right]}\;(m_{1},m_{2})$ and by offsetting the coordinate $u_{2}$ by 15 pixels.

Because the coplanarity of these rays cannot be guaranteed (skew rays case), a better triangulation approximation while considering coaxial misalignments is to find the midpoint of their common perpendicular line segment (as attempted in [23]). As illustrated in Figure 16, we define the common perpendicular line segment $\bar{G_{1}G_{2}}$ as the parametrized vector $v_{1⊥2}=\lambda_{1⊥2}{\hat{v}}_{1⊥2}$, for the unit vector normal to the back-projected rays, $v_{1}$ and $v_{2}$, such that:

(90) $$\begin{array}{c} {\hat{v}}_{1⊥2}=\frac{v_{1}⊗v_{2}}{||v_{1}⊗v_{2}||} \end{array}$$

If the rays are not parallel ($||v_{1}⊗v_{2}||\neq 0$), we can compute the “exact” solution, $\lambda ={\left[\begin{array}{ccc} \lambda_{G_{1}}, & \lambda_{G_{2}}, & \lambda_{1⊥2} \end{array}\right]}^{T}$, of the well-determined linear matrix equation

(91) $$\begin{array}{cc} V\lambda & =b\;,\;\text{where}\;V=\left[\begin{array}{ccccc} v_{1}, & & -v_{2}, & & {\hat{v}}_{1⊥2} \end{array}\right]\;\text{and}\;b={}^{\left[C\right]}\;f_{2}-{}^{\left[C\right]}\;f_{1} \end{array}$$

It follows that the location of the midpoint $P_{w_{G}}$ on the common perpendicular $v_{1⊥2}$ with respect to the common frame $\left[C\right]$ is

(92) $$\begin{array}{cc} {}^{\left[C\right]}\;p_{w_{G}} & ={}^{\left[C\right]}\;f_{1}+\lambda_{G_{1}}{}^{\left[F_{1}\right]}\;v_{1}+\frac{1}{2}\lambda_{1⊥2}{}^{\left[G_{1}\right]}\;{\hat{v}}_{1⊥2} \end{array}$$

#### 5.2.2. Range Variation

**Figure 17:**
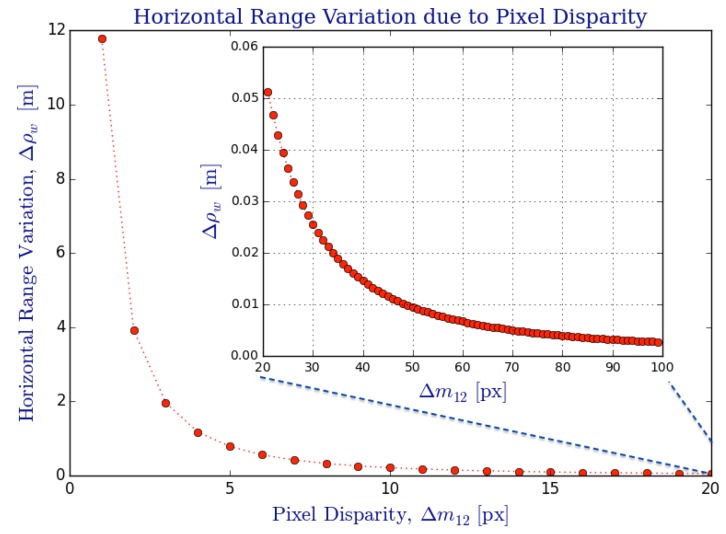
Variation of horizontal range, $\Delta \rho_{w}$, due to change in pixel disparity $\Delta m_{12}$ on the omnidirectional image, $\left[I\right]$. There exists a “nonlinear & inverse” relation between the change in depth from triangulation ($\Delta \rho_{w}$) and the number of disparity pixels ($\Delta m_{12}$) available from the omnistereo image pair $\left(\left[I_{1}\right],\left[I_{2}\right]\right)$, which are exclusive subspaces of $\left[I\right]$.

Before we introduce an uncertainty model for triangulation (Section 5.3), we briefly analyze how range varies according to the possible combinations of pixel correspondences, ${}^{\left[I\right]}\;(m_{1},m_{2})$ on the image $\left[I\right]$. Here, we demonstrate how a radial variation of discretized pixel disparities, $\Delta m_{12}$, affects the 3D position of a point obtained from triangulation (Section 5.2). Figure 17 demonstrates the nonlinear characteristics of the variation in horizontal range, $\Delta \rho_{w}$, from the discrete relation between pixel positions ${}^{\left[I\right]}\;m_{i}$ and their respective back-projected (direction) rays obtained from $f_{\beta_{i}}$ and triangulated via function $f_{\Delta }$ defined in Equation (89). It can be observed that the horizontal range variation, $\Delta \rho_{w}$, increases quadratically as $\Delta m_{12}\to 1\mathrm{px}$, which is the minimum *discrete* pixel disparity, which provides a maximum horizontal range $\rho_{w,max}\approx \left[18,28\right]$m (computed analytically). The main plot of Figure 17 shows the small disparity values in the interval $\bar{\Delta m_{12}}=\left[1,20\right]\;\mathrm{px}$, whereas the subplot is a zoomed-in extension of the large disparity cases in the interval $\bar{\Delta m_{12}}=\left[20,100\right]\;\mathrm{px}$.

The current analysis is an indicative that triangulation error (e.g., due to false pixel correspondences) may have a severe effect on range accuracy that increases quadratically with distance as it can be appreciated with the 8 m variation on the disparity interval $\bar{\Delta m_{12}}=\left[1,2\right]\mathrm{px}$. Also, observe the example of Figure 20 for a reconstructed point cloud, where this range sensing characteristic is more noticeable for faraway points. In fact, the following uncertainty model provides a probabilistic framework for the triangulation error (uncertainty) that agrees with the current numerical claims.

### 5.3. Triangulation Uncertainty Model

Let $f_{P_{w}}$ be the vector-valued function that computes the 3D coordinates of point $P_{w_{G}}$ with respect to $\left[C\right]$ as the common perpendicular midpoint defined in Equation (92). We express this triangulation function component-wise as follows:

(93) $$\begin{array}{c} {}^{\left[C\right]}\;p_{w_{G}}\leftarrow f_{P_{w}}\left(m_{12}\right)\left[\begin{array}{c} f_{x_{w}}\left(m_{12}\right)\\ f_{y_{w}}\left(m_{12}\right)\\ f_{z_{w}}\left(m_{12}\right) \end{array}\right] \end{array}$$

where $m_{12}=\left[u_{1},v_{1},u_{2},v_{2}\right]$ is composed by the pixel coordinates of the correspondence ${}^{\left[I\right]}\;(m_{1},m_{2})$ upon which to base the triangulation (Section 5.2).

Without loss of generality, we model a *multivariate Gaussian* uncertainty model for triangulation, so that the position vector ${}^{\left[C\right]}\;p_{w_{G}}$ of any world point is centered at its mean ${}^{\left[C\right]}\;\mu_{f_{P_{w}}}$ with a 3×3 covariance matrix $\Sigma_{f_{P_{w}}}$:

(94) $$\begin{array}{cc} {}^{\left[C\right]}\;\mu_{f_{P_{w}}}=\left[\begin{array}{c} x_{w}\\ y_{w}\\ z_{w} \end{array}\right], & \;\Sigma_{f_{P_{w}}}=\left[\begin{array}{ccccc} \sigma_{f_{x_{w}}}^{2} & & \sigma_{f_{x_{w}}}\sigma_{f_{y_{w}}} & & \sigma_{f_{x_{w}}}\sigma_{f_{z_{w}}}\\ \sigma_{f_{x_{w}}}\sigma_{f_{y_{w}}} & & \sigma_{f_{y_{w}}}^{2} & & \sigma_{f_{y_{w}}}\sigma_{f_{z_{w}}}\\ \sigma_{f_{x_{w}}}\sigma_{f_{z_{w}}} & & \sigma_{f_{y_{w}}}\sigma_{f_{z_{w}}} & & \sigma_{f_{z_{w}}}^{2} \end{array}\right] \end{array}$$

**Figure 18:**
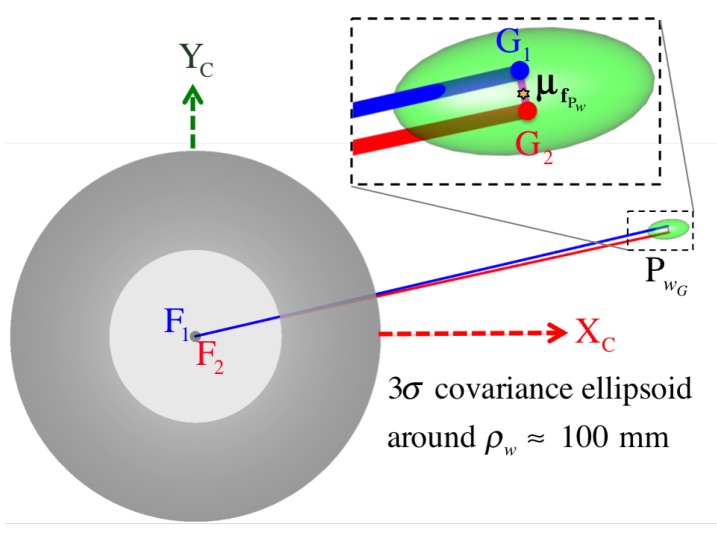
Top-view of the three-sigma level ellipsoid for the triangulation uncertainty of a pixel pair ${}^{\left[I\right]}\;(m_{1},m_{2})$ with an assumed standard deviation $\sigma_{px}=1\;\mathrm{px}$.

**Figure 19:**
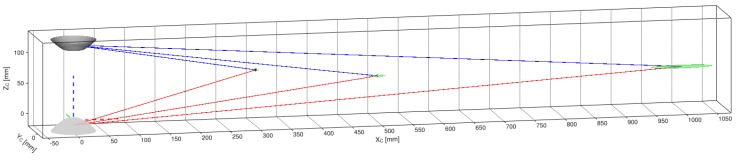
Uncertainty ellipsoids for triangulated points at ranges $\rho_{w}\approx \left\{0.3,0.5,1.0\right\}$m.

However, since $f_{P_{w}}$ is a non-linear vector-valued function, we linearize it by approximation to a first-order Taylor expansion and we use its Jacobian matrix to propagate the uncertainty (covariance) as in the linear case as follows:

(95) $$\begin{array}{c} \Sigma_{f_{P_{w}}}=J_{f_{P_{w}}}\;\Omega_{m_{12}}\;{J_{f_{P_{w}}}}^{T} \end{array}$$

where the 3×4 Jacobian matrix for the triangulation function is

(96) $$\begin{array}{c} J_{f_{P_{w}}}=\left[\begin{array}{cccc} \frac{\partial f_{x_{w}}}{\partial u_{1}} & \frac{\partial f_{x_{w}}}{\partial v_{1}} & \frac{\partial f_{x_{w}}}{\partial u_{2}} & \frac{\partial f_{x_{w}}}{\partial v_{2}}\\ \frac{\partial f_{y_{w}}}{\partial u_{1}} & \frac{\partial f_{y_{w}}}{\partial v_{1}} & \frac{\partial f_{y_{w}}}{\partial u_{2}} & \frac{\partial f_{y_{w}}}{\partial v_{2}}\\ \frac{\partial f_{z_{w}}}{\partial u_{1}} & \frac{\partial f_{z_{w}}}{\partial v_{1}} & \frac{\partial f_{z_{w}}}{\partial u_{2}} & \frac{\partial f_{z_{w}}}{\partial v_{2}} \end{array}\right] \end{array}$$

and the 4×4 covariance matrix of the pixel arguments being

(97) $$\begin{array}{c} \Omega_{m_{12}}=\sigma_{px}^{2}I_{4} \end{array}$$

where we assume $\sigma_{px}=1\;\mathrm{px}$ for the standard deviation of each pixel coordinate in the *discretized* pixel space. The complete symbolic solution of $\Sigma_{f_{P_{w}}}$ is too involved to appear in this manuscript. However, in Figure 18, we show the top-view of the covariance ellipsoid drawn at a three-$\sigma_{f_{P_{w}}}$ level for a point triangulated nearly around $\rho_{w}\approx 100\mathrm{mm}$. Figure 19 visualizes uncertainty ellipsoids drawn at a one-$\sigma_{f_{P_{w}}}$ level for several triangulation ranges. We refer the reader to the end of Section 6.3 where we validate the safety of this 1 pixel deviation assumption through experimental results using subpixel precision.

## 6. Experiment Results

In this section, we demonstrate the capabilities of the omnistereo sensor to provide 3D information either as dense point clouds or as for the registration of sparse 2D features and 3D points. We also evaluate the precision of both projection and triangulation of a few detected corners from a chessboard whose various 3D poses are given as ground-truth.

### 6.1. Dense 3D Point Clouds

**Figure 20:**
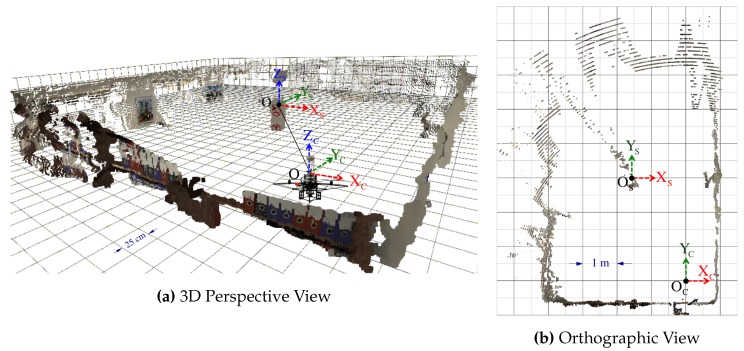
A 3-D dense point cloud computed out of the synthetic model that rendered the omnidirectional image shown in Figure 2b. Pixel correspondences are established via the panoramic depth map visualized in Figure 15. The 3D point triangulation implements the common perpendicular midpoint method indicated in Section 5.2.1. The position of the omnistereo sensor mounted on the quadrotor is annotated as frame $\left[C\right]$ with respect to the scene’s coordinates frame $\left[S\right]$. (**a**) 3D visualization of the point cloud (the quadrotor with the omnistereo rig has been added for visualization only); (**b**) Orthographic projection of the point cloud to the XY-plane of the visualization grid.

By implementing the process described in Section 5, we begin by visualizing the dense point-cloud obtained from the omnidirectional synthetic image given in Figure 2b, whose actual size is 1280 × 960 pixels. The associated panoramic images, $\left[\Xi_{i}\right]$, were obtained using function $h_{\Xi_{i}}$ defined in Equation (85) and are shown in Figure 15. Pixel correspondences $\left({}^{\left[\Xi_{1}\right]}\;m_{1},{}^{\left[\Xi_{2}\right]}\;m_{2}\right)$ on the panoramic representations are mapped via $h_{\Xi_{i}}$ into their respective image positions ${}^{\left[I\right]}\;(m_{1},m_{2})$. Then, these are triangulated with ${}^{\left[C\right]}\;f_{P_{w}}$ given in Equation (93), resulting in the set (cloud) of color 3D points $P_{\Delta }$ visualized in Figure 20. Here, the synthetic scene (Figure 2a) is for a room 5.0 m wide (along its X-axis), 8.0 m long (along its Y-axis), and 2.5 m high (along its Z-axis). With respect to the scene center of coordinates, $\left[S\right]$, the catadioptric omnistereo sensor, $\left[C\right]$, is positioned at ${}_{\left[C\right]}^{\left[S\right]}\;t={\left[\begin{array}{ccc} 1.60, & -2.85, & 0.16 \end{array}\right]}^{T}$ in meters.

**Figure 21:**
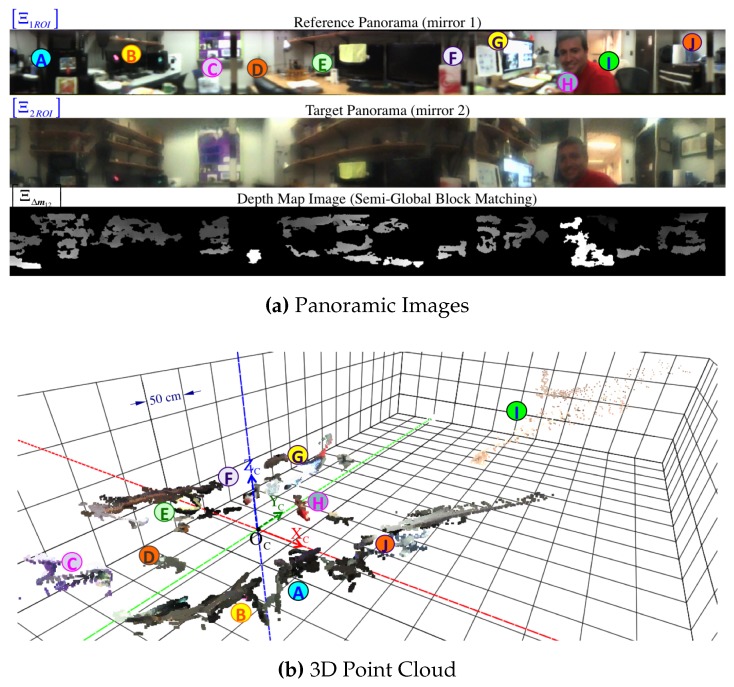
Real-life experiment using the 37mm-radius prototype and a single 2592 × 1944 pixels image where the rig was positioned in the middle of the room observed in Figure 13a. Some landmarks of the scene are annotated as following: Ⓐ appliances, Ⓑ monitors and shelf, Ⓒ back wall, Ⓓ chair, Ⓔ monitors and shelf,Ⓕ book, Ⓖ monitors, Ⓗ person, Ⓘ hallway, Ⓙ supplies. For the point cloud, the grid size is 0.50 m in all directions and points are thickened for clarity.

We also present results from a real experiment using the prototype described in Section 4.3.2 and shown in Figure 13a. The panoramic images and dense point cloud shown in Figure 21 are obtained by implementing the pertinent functions described throughout this manuscript and by holding the SVP assumption of an ideal configuration. We provide these qualitative results as preliminary proof of concept for the proposed sensor after employing a calibration procedure based on the generalized unified model proposed in [37].

### 6.2. Sparse 3D Points from Features

**Figure 22:**
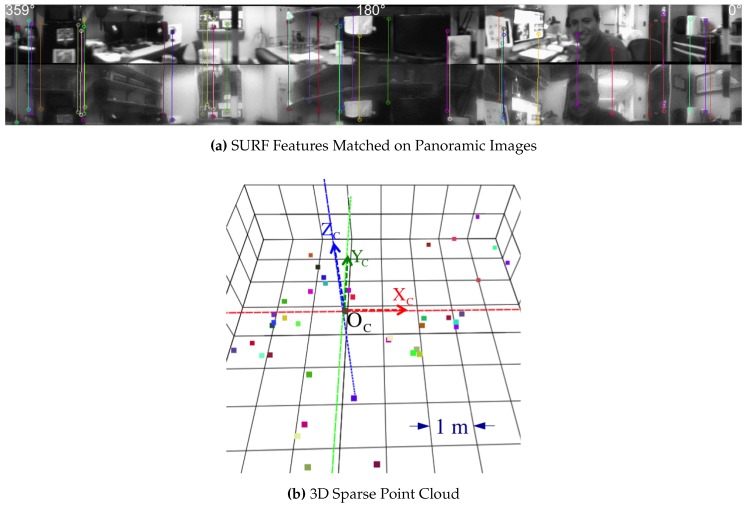
Sparse point correspondences for the real-life image from Figure 13b. Point correspondences are identifiable by random colors that persist in both the panoramic image and the respective triangulated 3D points (scaled-up for visualization).

Using the SURF feature detector and descriptors [38], Figure 22 demonstrates 44 correct matches that are triangulated with Equation (93). Sparse 3D points can be useful for applications of visual odometry where the sensor changes poses and those registered point features can be matched against new images. Please, refer to [39] for a tutorial on visual odometry.

### 6.3. Triangulation Evaluation

#### 6.3.1. Evaluation of Synthetic Rig

Due to the unstructured nature of the dense point clouds previously discussed, we proceed to triangulate sets of sparse 3D points whose positions with respect to the omnistereo sensor camera frame, $\left[C\right]$, are known in advance. We synthesize a calibration chessboard pattern $\left[G\right]$ containing m×n square cells for various predetermined poses ${}_{\left[G\right]}^{\left[C\right]}\;T_{h}$. Since the sensor is assumed to be rotationally symmetric, it suffices to experiment with groups of L=4 chessboard patterns situated at a given horizontal range. A total of Lmn 3D points are available for each range group. Each corner point’s position ${}^{\left[C\right]}\;p_{j}$ is found with respect to $\left[C\right]$ via the frame transformation ${}^{\left[C\right]}\;p_{j,g}={}_{\left[C_{g}\right]}^{\left[C\right]}\;T_{h}{}^{\left[C_{g}\right]}\;p_{j}$ for all indices j∈{1,…,mn},g∈{1,…,L}.

**Figure 23:**
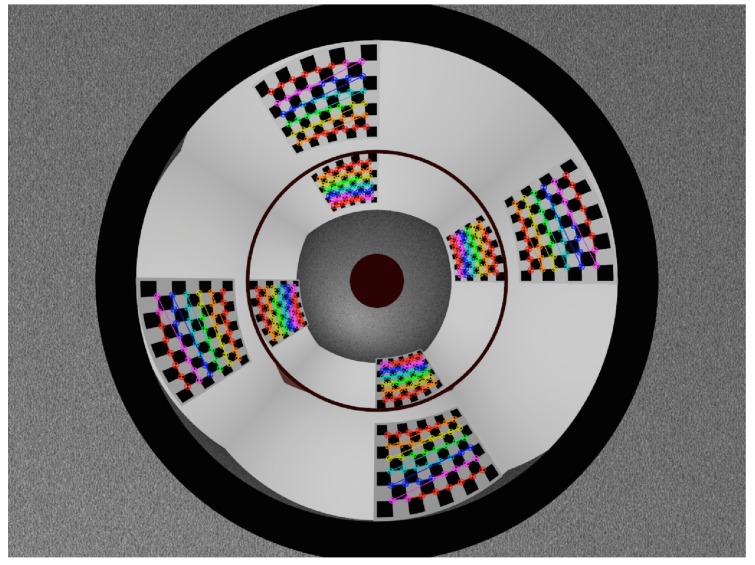
Example of sparse point correspondences detected with subpixel precision from corners on the chessboard patterns around the omnistereo sensor. The size of the rendered images for this experiment is 1280 × 960 pixels. For this example’s patterns, the square cell size is 140mm. The RMSE for this set of points at ${}^{\left[C\right]}\;\rho_{G}=2m$ is approximately 15mm (Table 4).

Figure 23 shows the set of detected corner points on the image from the group of patterns set to a range of ${}^{\left[C\right]}\;\rho_{G}=2m$. We adjust the pattern’s cell sizes accordingly so its points can be safely discerned by an automated corner detector [35]. We systematically establish correspondences of pattern points on the omnidirectional image, and proceed to triangulate with Equation (93). For each range group of points, we compute the root-mean-square of the 3D position errors (RMSE) between the observed (triangulated) points ${}^{\left[C\right]}\;{\tilde{p}}_{j}\leftarrow f_{P_{w}}\left({\tilde{m}}_{1},{\tilde{m}}_{2}\right)$ and the true (known) points ${}^{\left[C\right]}\;p_{j}$ that were used to describe the ray-traced image. Table 4 compiles the RMSE results and the standard deviation (SD) for some group of patterns whose frames $\left[G_{g}\right]$, are located at specified horizontal ranges ${}^{\left[C\right]}\;\rho_{G}\in [0.25,8.0]$m away from $\left[C\right]$.

We notice that for all the 3D points in the synthetic patterns, we obtained an average error of 0.1px with a standard deviation ${\tilde{\sigma }}_{px}=0.05\;\mathrm{px}$ for the subpixel detection of corners on the image versus their theoretical values obtained from $f_{\varphi_{i}}$ defined in Equation (36). This last experiment helps us validate the pessimistic choice of $\sigma_{px}=1\;\mathrm{px}$ for the discrete pixel space in the triangulation uncertainty model proposed in Section 5.3.

#### 6.3.2. Evaluation of Real-Life Rig

**Figure 24:**
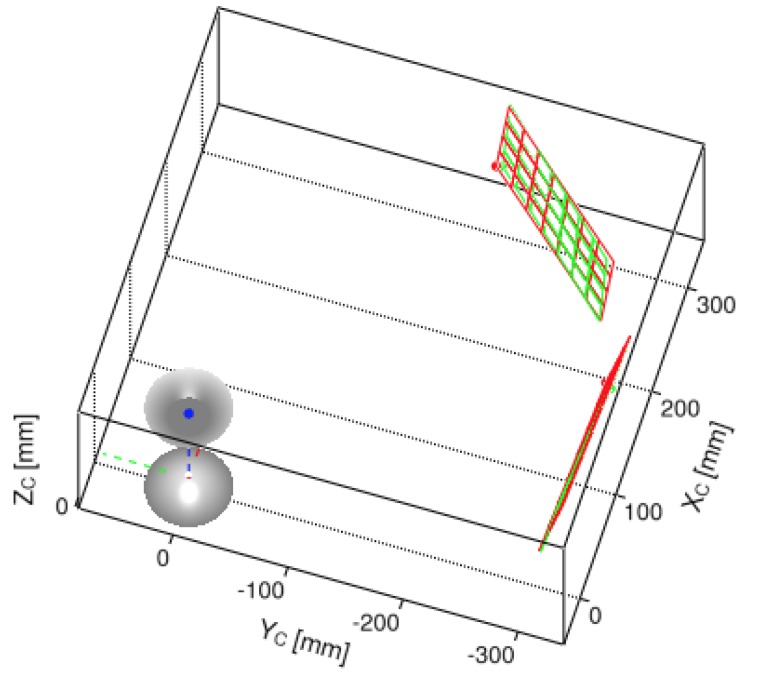
Visualization of estimated 3D poses for some chessboard patterns using the real-life omnistereo rig. Color annotations: ground-truth poses (green), estimated triangulated poses (red).

The following experiment uses L=5 different poses of a real chessboard pattern with 5×8 corner points where the square cell size is 24mm. As done in Section 6.3.1, the evaluated error is the Euclidean norms between the triangulated points and the ground-truth positions of the chessboard posses captured via a motion capture system. The RMSE for all projected points in this set of chessboard patterns is 2.5 pixels with a standard deviation of 1.5 pixels. The RMSE for all triangulated points in this set is 3.5mm with a standard deviation of 1.4mm. Figure 24 visually confirms the proximity of the triangulated chessboard poses against the ground-truth pose information.

## 7. Discussion and Future Work

The portable aspect of the proposed omnistereo sensor is one of its greatest advantages, as discussed in the introduction section. The total weight of the big rig using 37mm-radius mirrors is about 550g, so it can be carried by the AscTec Pelican quadrotor under its payload limitations of 650g. The mirror profiles maximize the stereo baseline while obeying the various design constraints such as size and field of view. Currently, the mirrors are custom-manufactured out of brass using CNC machining. However, it is possible to reduce the system’s weight dramatically by employing lighter materials.

In reality, it is almost impossible to assemble a perfect imaging system that fulfills the SVP assumption and avoids the triangulation uncertainty studied in Section 5.3 on top of the error already introduced by any feature matching technique. The coaxial misalignment of the folded mirrors-camera system, defocus blur of the lens, and the inauspicious glare from the support tube are all practical caveats we need to overcome for better 3D sensing tasks. As described in the text for the real-life rig, we have avoided the traditional use of a support cylinder in order to workaround the cross-reflections and glare issues. Possible vibrations caused by the robot dynamics are reduced by vibration pads placed on the sensor-body interface. Details about our tentative calibration method for vertically-folded omnistereo systems has not been included in the current study since we would like the reader’s attention to be devoted to the sensor characteristics defended by this analysis.

Our ongoing research is also focusing on the development of efficient software algorithms for real-time 3D pose estimation from point clouds. Bear in mind that all the experimental results demonstrated in this manuscript rely upon a single camera snapshot. We understand that the narrow vertical field-of-view where stereo vision operates is a limiting factor for dense scene reconstruction from a single image, so we have also considered non-optimal geometries for the quadrotor’s view. In fact, increasing the region of interest for stereo (SROI) while maintaining the wide baseline implies an enlargement of each mirror’s radius. We believe that our omnidirectional system is more advantageous than forward-looking sensors because it can provide a robust pose estimation by extracting 3D point features from all around the scene at once. As in our past work [24], fusing multiple modalities (e.g., stereo and optical-flow) is a possibility in order to resolve the scale-factor problem inherent while performing structure from motion over the non-stereo regions of each mirror (near the poles).

In this work, we performed an extensive study of the proposed omnistereo sensor’s properties, such as its spatial resolution and triangulation uncertainty. We validated the projection accuracy of the synthetic model (the ideal case) where 3D points in the world are given exactly. In order to validate the precision of the real sensor, we require a perfectly constructed and assembled device so point projections can be accepted as the ultimate truth. This is hard to achieve at a low-cost prototyping stage. Although we acquired ground-truth 3D points via a position capture system alone, we deem this insufficient to validate the imaging accuracy of the real sensor because the precision of the calibration method is truly what is being accounted for. For reproducibility purposes, source code is available for the implementation of the theoretical omnistereo model, optimization, plots and figures presented in this analysis [40].

## Figures and Tables

**Table 1:** Optimal System Design Parameters.

Parameter	Big Rig	Small Rig
$b=\max f_{b}\left(\theta^{*}\right)$	131.61	108.92
$r_{sys}\left[\mathrm{mm}\right]$	37.0	28.0
$c_{1}\left[\mathrm{mm}\right]$	123.49	104.59
$c_{2}\left[\mathrm{mm}\right]$	241.80	204.34
d[mm]	233.68	200.00
$k_{1}$	5.73	6.88
$k_{2}$	9.74	11.47

**Table 2:** By-product Length Parameters.

Parameter	Big Rig	Small Rig
$r_{ref}\left[\mathrm{mm}\right]$	17.23	11.74
$r_{cam}\left[\mathrm{mm}\right]$	7	7
$h_{sys}\left[\mathrm{mm}\right]$	150.00	120.00

**Table 3:** Near Vertices of the SROI for the Big Rig.

Vertex	${}^{\left[C\right]}\;\rho_{w}$ [mm]	${}^{\left[C\right]}\;z_{w}$ [mm]
$P_{ns_{high}}$	93.5	144.4
$P_{ns_{mid}}$	65.2	98.4
$P_{ns_{low}}$	763.4	-170.3

**Table 4:** Results of RMSE from Synthetic Triangulation Experiment.

${}^{\left[C\right]}\;\rho_{G}$ [m]	RMSE [mm]	SD [mm]
0.25	0.46	0.31
0.50	1.20	0.71
1.0	4.62	2.55
2.0	14.85	9.06
4.0	57.67	31.34
8.0	219.09	129.92

## Appendix A. Symbolic Notation

### Abbreviations

$P_{i}$: a point $\in R^{3}$ where post-subscript *i* as a unique identifier.
$\left[A\right]$: a reference frame or image space with origin $O_{A}$.
${}^{\left[A\right]}\;p_{i}$: The position vector of $P_{i}$ with respect to reference frame $\left[A\right]$.
${}^{\left[A\right]}\;p_{i,h}$: for homogeneous coordinates.
${}^{\left[I\right]}\;m_{i}$: a 2D point or pixel position on image frame $\left[I\right]$.
$\left\|p_{i}\right\|$: the magnitude (Euclidean norm) of $p_{i}$.
$\hat{q}$: A unit vector so $||\hat{q}||=1$.
$M_{i}$: a 3×3 matrix, or $M_{i,h}$ in homogeneous coordinates.
$f_{s}$: a scalar-valued function that outputs some *s*.
$f_{v}$: a vector-valued function for the computation of v.

All coordinate systems obey the right-hand rule unless otherwise indicated.
